# Discrimination of Steatotic and Non-Steatotic Chemicals Through Transcriptome Analysis in Primary Human Hepatocytes

**DOI:** 10.3390/ijms27093825

**Published:** 2026-04-25

**Authors:** Christina A. Cramer von Clausbruch, Marcha Verheijen, Giulia Callegaro, Jonathan H. Freedman, Rita Ortega-Vallbona, Martina Palomino-Schätzlein, Florian Caiment, Carsten Weiss

**Affiliations:** 1 Institute of Biological and Chemical Systems—Biological Information Processing, Karlsruhe Institute of Technology (KIT), 76344 Eggenstein-Leopoldshafen, Germany; c.cramer-von-clausbruch@gmx.de; 2 Department of Translational Genomics, Research Institute for Oncology and Reproduction, Maastricht University, 6229 MD Maastricht, The Netherlands; verheijen.m@gmail.com (M.V.); florian.caiment@maastrichtuniversity.nl (F.C.); 3 Division of Cell Systems and Drug Safety, Leiden Academic Centre for Drug Research, Leiden University, Einsteinweg 55, 2333 CC Leiden, The Netherlands; g.callegaro@lacdr.leidenuniv.nl; 4 Centre for Environmental Research and Justice and School of Biosciences, University of Birmingham, Birmingham B15 2TT, UK; jon.freedman@wormtox.org; 5 ProtoQSAR SL, Parque Tecnológico de Valencia, 46980 Paterna, Spain; rortega@protoqsar.com (R.O.-V.); mpalomino@protoqsar.com (M.P.-S.); 6 MolDrug AI Systems SL, 46018 Valencia, Spain

**Keywords:** systems toxicology, new approach methods, adverse outcome pathways

## Abstract

Steatosis, characterized by excessive fat accumulation in the liver, is a significant precursor to chronic liver disease and hepatocarcinoma. This condition is influenced by multiple contributing factors such as obesity, alcohol consumption, and exposure to chemicals or drugs. Systems biology approaches including transcriptomics and metabolomics can aid in grouping chemicals according to their mode of action. In this study, we analyze transcriptomic and metabolomic data from primary human and transformed hepatocytes, respectively, to differentiate between steatotic and non-steatotic chemicals. Rather than assessing each steatotic compound individually, we pooled several steatotic chemicals in order to minimize compound-specific noise and better identify features associated with the underlying process of steatosis. Differential gene expression analysis revealed established mechanisms involved in steatosis, consistent with the recently updated adverse outcome pathway. Likewise, metabolomic data enabled clear discrimination between steatotic and non-steatotic chemicals. These findings highlight the potential of omics technologies to support chemical grouping based on insights into the molecular mechanisms that drive steatosis development.

## 1. Introduction

Steatosis, characterized by abnormal hepatic fat deposition, is a multifactorial condition with profound implications for human health. It can arise due to an array of factors, including obesity, alcohol consumption, chemical exposure and certain pharmaceuticals. The accumulation of fatty acids within liver cells disrupts normal liver function. The accumulation can progress into a range of associated diseases, most notably Non-Alcoholic Fatty Liver Disease (NAFLD), which has been recently redefined as metabolic dysfunction-associated steatotic liver disease (MASLD) [1,2,3]. MASLD is not solely defined by the presence of hepatic steatosis but is commonly associated with additional metabolic conditions such as obesity, type 2 diabetes, hypertension, and hypertriglyceridemia. The disease comprises several stages and subcategories. The earliest stage is simple steatosis, characterized by lipid accumulation in hepatocytes without significant inflammation or fibrosis. Metabolic dysfunction–associated steatohepatitis (MASH) represents a more severe form of MASLD and is marked by hepatic inflammation, hepatocellular injury, and varying degrees of fibrosis. As the disease progresses, fibrosis can advance to cirrhosis, a condition characterized by extensive scar tissue formation and impaired liver function. In some cases, cirrhosis may further progress to hepatocellular carcinoma (HCC), the most severe stage of MASLD, in which malignant liver tumours develop [4]. MASLD is currently the most prevalent cause of chronic liver disease, affecting nearly 32% of the global population [5,6,7,8].

Steatosis is a complex disease and is driven by a broad network of interconnected molecular pathways. Central to steatosis development is the dysregulation of lipid metabolism, particularly the imbalance between lipid uptake, synthesis, and export. Excessive lipid accumulation induces oxidative stress, triggering the release of pro-inflammatory cytokines and promoting chronic inflammation, involving nuclear factor (NF) -κB. This inflammatory cascade contributes to progressive liver damage. At the molecular level, several critical events and key players, such as the occurrence of insulin resistance, and peroxisome proliferator-activated receptor (PPAR) and (mTOR) signalling, have been recognized as important contributors to steatosis progression [2,9,10,11].

Specific molecular mechanisms and markers have been identified in hepatocytes for some of the key processes, such as proteins involved in fatty acid uptake (CD36, FATP2) and lowered lipid export (ApoB), resulting in lipid accumulation. Insulin resistance has been linked to reduced AKT phosphorylation and increased SREBP1 activity enhancing lipogenesis. In addition, impaired mitochondrial function and enhanced oxidative stress are connected to impaired beta-oxidation (involving PPARs), reduced ATP levels and elevated reactive oxygen species resulting in hepatocyte injury and death. Furthermore, endoplasmatic reticulum (ER) stress, via the ATF4 and unfolded protein response pathways, exacerbates lipid accumulation and inflammatory signalling [4].

Although MASLD is increasingly recognized as a multiple-hit disease, the interplay among its contributing factors remains incompletely understood. Furthermore, there is currently no standardized screening or treatment protocol. While guidelines for the identification and staging of steatosis, as well as clinical practical guidelines for symptomatic management are available, reliable biomarkers and therapeutic targets for early diagnosis and intervention remain to be established [6,7,8,12,13,14,15].

Although animal model-derived data have long been considered the gold standard in both regulatory and research settings, there is growing interest in developing more ethically acceptable, cost-effective, and time-efficient alternatives. In this context, New Approach Methodologies (NAMs) are being developed in alignment with the 3R principle (replacement, reduction, and refinement of animal use). These include in silico modelling, high-throughput screening assays, toxicogenomics, and machine learning systems, among other innovative tools [16,17,18,19]. To deepen our understanding of the modes of action of chemicals, molecular approaches, such as transcriptomics, proteomics and metabolomics, offer powerful means to explore biological pathways and molecular responses triggered by individual compounds or even complex mixtures [20,21,22,23].

Adverse outcome pathways (AOPs) and AOP networks emerge as a new concept to structure and leverage the wealth of existing molecular and phenotypic data. They aim to provide mechanistic knowledge to better understand diseases such as steatosis. AOPs are designed to systematically represent critical biological processes and how they are linked to adverse health outcomes, particularly in the context of chemical exposure [24].

AOPs are not merely linear pathways but are more complex and multifactorial. They causally link multiple events, starting with molecular initiating events (MIEs), which represent the apical chemical interaction with a molecular target. From MIEs, the pathway proceeds through a series of key events (KEs), which reflect different biological processes that might occur at various levels of organization, such as cellular or tissue responses. These KEs build upon one another, with the accumulation of noxious changes that lead to the final adverse outcome (AO), which is typically an observable and measurable detrimental effect on health. A simplified overview is shown in Supplemental Figure S1 [25].

Importantly, AOPs are often dynamic and non-linear, influenced by factors like genetic susceptibility and environmental context. AOPs can be assembled in networks, where multiple pathways can converge on a shared adverse outcome. Although still evolving, with ongoing harmonization and updates, AOPs provide valuable insight into complex toxicological mechanisms, offering a more comprehensive understanding of chemical-induced diseases and improving predictive models for regulatory risk assessments [25,26,27,28].

The current study was conducted as part of the ASPIS Cluster, a collaborative initiative comprising the H2020-funded EU projects ONTOX [28], RISK-HUNT3R [29] and PrecisionTox [30], which collectively aim to pioneer novel strategies for sustainable, animal-free, and reliable chemical risk assessment [29].

Here, we analyzed publicly available transcriptomic data from primary human hepatocytes to identify shared molecular signatures associated with chemical-induced steatosis. By examining gene expression changes after short-term exposure to different concentrations of steatotic versus non-steatotic chemicals, we identified critical patterns indicative of early steatosis development. Alterations in the transcriptome were compared to differences in the metabolome to comprehensively assess the impact of steatotic chemicals. The primary aim of this study was not to define new features of steatosis through the interrogation of omics datasets, but rather to investigate whether known steatotic and non-steatotic chemicals can be distinguished based on their omics profiles. Such separation based on omics profiling may support the grouping of chemicals into different hazard categories. Subsequently, the identified signatures were compared with the existing framework and adverse outcome pathways (AOPs) of steatosis to determine whether the steatotic chemicals analyzed indeed affect genes and pathways relevant to steatosis. Verhoeven et al. [27] have made significant progress in defining the quantitative weight of evidence for steatosis AOP networks, and recent studies such as Bwanya et al. [30] have applied machine learning (ML) models, including Support Vector Machines (SVM), in predictive toxicology. ML models such as SVMs are powerful tools for binary classification and toxicity prediction, as they rely on the accumulation of multiple subtle trends in gene regulation to classify chemicals. While effective for discrimination, these approaches are often more difficult to interpret from a biological perspective. In contrast, our approach combines gene set enrichment analysis (GSEA), differential expression analysis, and data visualization and enrichment analysis of transcriptomics data (DEVEA) with metabolomics to provide mechanistic insights into modes of action. By specifically comparing pooled steatotic chemicals with non-steatotic controls, we directly map transcriptomic alterations to established key events in the steatosis AOP. Our goal is therefore not solely classification, but rather the identification of shared molecular signatures and interconnected pathways relevant to the development of steatosis.

Differential gene expression analysis revealed signatures that discriminated steatotic from non-steatotic chemicals. Additionally, some of these pathways were aligned with critical events described in the established AOP for steatosis [27]. Thus, specific transcriptomic alterations may serve as predictive markers for grouping chemicals and assessing their potential to induce steatosis, to support future chemical hazard and risk assessment.

## 2. Results and Discussion

### 2.1. Analysis of Differentially Expressed Genes in Response to Steatotic Chemicals

**Figure 1:**
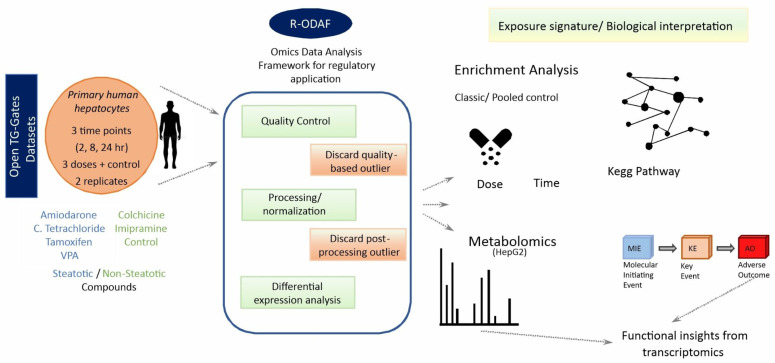
Overview of study design.

We employed the Omics Data Analysis Framework for Regulatory Application (R-ODAF), a robust tool for OMICs analysis, providing essential pre-processing steps and yielding reproducible data [31,32]. Selected microarray datasets were processed by R-ODAF to identify differentially expressed genes (DEGs) regulated by steatotic chemicals in primary human hepatocytes (PHHs). The data was sourced from the Toxicogenomics Project—Genomics Assisted Toxicity Evaluation system (TG-GATEs), which evaluated up to 170 different chemicals in primary human and rat hepatocytes as well as in in vivo rat models, generating transcriptomic profiles from over 20,000 microarrays [33,34]. Microarray data was available for four steatotic (amiodarone, carbon tetrachloride, tamoxifen and valproic acid) and for two non-steatotic chemicals (colchicine and imipramine). For metabolomics studies (see 2.4), datasets were available for six steatotic (amiodarone, cyclosporine A, fialuridine, tamoxifen, tetracycline, and valproic acid) and six non-steatotic compounds (amikacin, citrate, colchicine, cumene hydroperoxide, gentamicin, and imipramine). Utilizing the human data only, the number of DEGs was calculated by comparison of the pooled samples exposed to steatotic chemicals with untreated controls (T/CC). A simplified overview of the workflow and the study design is provided in Figure 1.

DEG analysis was performed across various time points and doses with *p*-value (0.05) where an adjusted *p*-value (0.05) led to no results (Supplemental Figure S2A, left panel). At two hours after treatment, a dose-dependent increase in down-regulated genes could be observed. In contrast, at later time points (8 and 24 h), a marked up-regulation of genes was detected at the low dose.

In addition to the conventional approach of comparing hepatocytes treated with steatotic chemicals to untreated controls, we explored a novel strategy to more effectively discriminate chemicals with known steatotic properties from non-steatotic chemicals. To this end, the relative transcript levels detected in cells exposed to steatotic chemicals were normalized to the average levels measured in a pooled control group comprising untreated cells and cells treated with non-steatotic compounds (T/PCs). Indeed, at later time points (8 and 24 h), this method revealed a greater number of DEGs at both medium and high doses (Supplemental Figure S2A, right panel). This observation suggests that steatotic chemicals induce more genes relative to the pooled control, likely because non-steatotic chemicals tend to down-regulate these genes, thereby amplifying the relative differences in gene expression (Supplemental Figure S3).

To address the impact of time on gene expression, we pooled all samples treated with the three doses and analyzed them across the different time points (Supplemental Figure S2B, left panel). The analysis showed an increased number of DEGs at the later time points of 8 and 24 h. Consistent with earlier findings, including samples treated with non-steatotic chemicals, the detection of DEGs was enhanced (Supplemental Figure S2B, right panel). To consider the influence of dose, all samples treated at the three time points were pooled and analyzed at the different doses (Supplemental Figure S2C). The highest number of DEGs could be identified at the low dose when untreated cells were used as the control (T/CC). As seen before, the inclusion of samples treated with non-steatotic chemicals increased the number of DEGs, particularly at medium and high doses (T/PC). The statistical analysis did not reveal significantly de-regulated DEGs in the case of a separate analysis of different time points and concentrations. Only for the dose- or time-dependent comparison of cells exposed to steatotic chemicals versus the pooled control group comprising untreated cells and cells treated with non-steatotic compounds (T/PC) were DEGs enriched at adjusted *p*-value 0.05 limits (marked by *).

### 2.2. Transcriptomics Identifies Pathways Impacted by Steatotic Chemicals with Relevance for the Established AOP for Steatosis

As gene-level analyses did not consistently discriminate between steatotic and non-steatotic exposures for all the different conditions, we performed gene set enrichment analysis using a pre-rank of genes by t-statistic. Employing GSEA (Broad Institute Inc.) allowed for pathway enrichment analysis with sound statistical filters for all datasets with a focus on pathways relevant for steatosis. An AOP specific for chemical-induced steatosis has been recently established and updated, outlining several critical molecular initiating and key events that lead to adverse outcomes ([27], Supplemental Figure S4).

**Figure 2:**
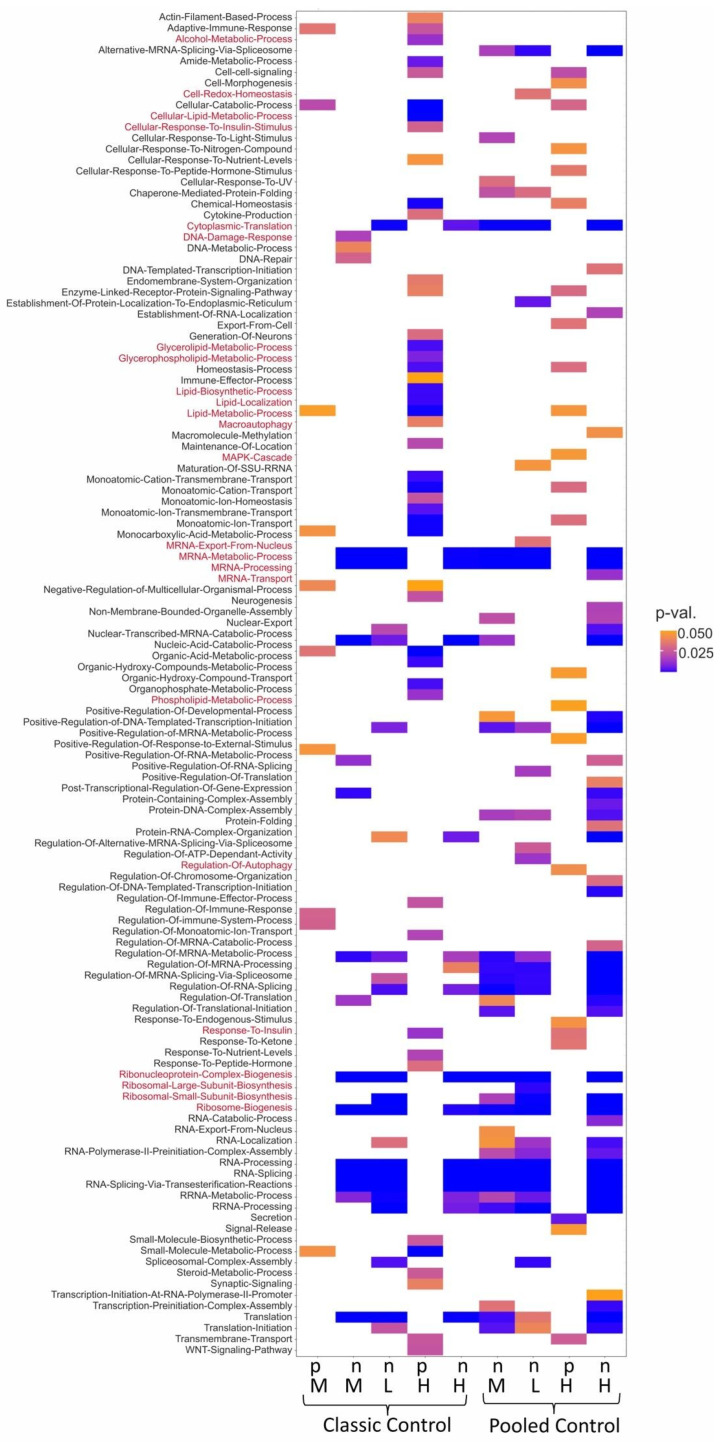
Dose-dependent enrichment analysis performed with GSEA identifies up- or down-regulated biological processes (GO terms) with relevance for steatosis. On (**left**) panel, identified pathways are shown based on the comparison of DEGs induced by steatotic compounds relative to untreated cells (classic control). On (**right**) panel, identified pathways are shown based on the comparison of DEGs induced by steatotic compounds relative to untreated cells and cells treated with non-steatotic compounds (pooled control). Shown are heatmaps, colour-coded according to *p*-value, with selected GO terms related to key events according to the AOP of steatosis (in red) with relevance for the adverse outcome pathway established for steatosis. (p = positive enrichment score, n = negative enrichment score, L = low dose, M = middle dose, H = high dose).

Shown in Figure 2 is the enrichment at each dose using untreated cells as the control (on the left) and the pooled control consisting of untreated cells and samples treated with non-steatotic chemicals (on the right). The database of gene ontology (GO) terms for biological processes proved to be most effective concerning retrieval of significantly enriched pathways. A broad range of GO terms could be identified including some with direct relevance for steatosis, such as lipid metabolism and processes linked to ER stress, which were up-regulated at the high dose and previously implicated in steatosis [27,28]. When samples treated with non-steatotic chemicals were included in the control group, overall pathway coverage increased slightly, particularly at the medium and high dose. The separate time-dependent analysis covered similar up-regulated pathways specifically at the late time point of 24 h (Supplemental Figure S5). Utilizing the databases WikiPathways and hallmark gene sets revealed much fewer pathways being affected in response to steatotic chemicals as compared to the GO knowledgebase. However, additional targets such as mTOR signalling, estrogen signalling and the unfolded protein response (UPR) could be identified (Supplemental Figures S6–S8). Thus, the selection of specific databases determines the breadth and specificity of the pathway analysis. As different complementary pathways were identified dependent on the database, leveraging the different databases provides a more comprehensive analysis. Although gene-level effects are modest and context-dependent as described above under Section 2.1, pathway-level analyses reveal biologically consistent responses that differentiate steatotic from non-steatotic exposures when dose and time are considered appropriately.

Nevertheless, for the dose- and time-dependent analysis of treated cells versus pooled controls (T/PCs) many statistically relevantly regulated genes were identified (Figure S2B,C). Therefore, we further investigated these DEGs and performed pathway enrichment analysis (PEA) with differential expression analysis, and the data visualization and enrichment analysis of transcriptomics data (DEVEA) tool.

**Figure 3:**
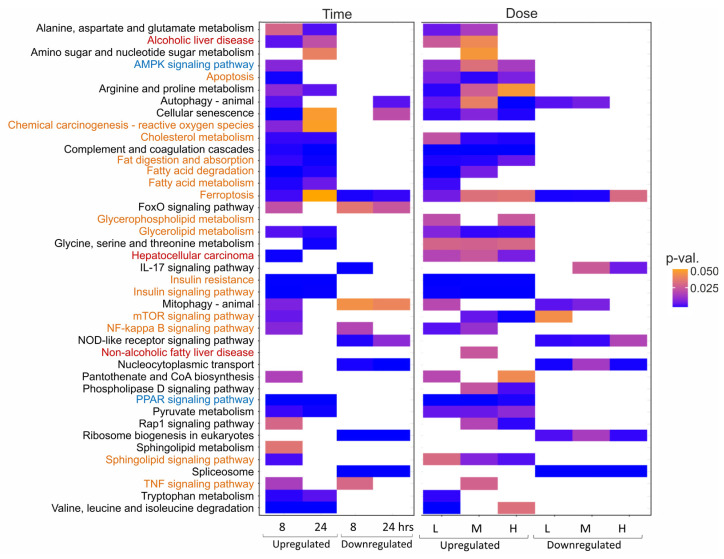
Pathway enrichment analysis (PEA) performed with DEVEA with relevance for steatosis dependent on time or dose. Identified pathways are shown based on the comparison of DEGs induced by steatotic compounds relative to untreated cells and cells treated with non-steatotic compounds (pooled control). (**left**) panel: PEA depending on time. All samples treated with the three doses were pooled and analyzed to depict the impact of time. (**right**) panel: PEA depending on dose. All samples treated at the three time points were pooled and analyzed to depict the impact of dose. (L = low dose, M = middle dose, H = high dose). Shown are heatmaps, colour-coded according to *p*-value, for a selection of steatosis-relevant pathways as defined by the respective AOP. Molecular initiating events (MIEs), key events (KEs) and adverse outcomes (AOs) are highlighted in blue, orange and red, respectively.

Considering the proposed and updated AOP for steatosis [27,28], we examined pathways related to molecular initiating events (e.g., PPAR and AMPK signalling), key events (e.g., mTOR signalling, TNF signalling, cholesterol and fatty acid metabolism, insulin resistance) and adverse outcomes (e.g., apoptosis, hepatocellular carcinoma, NAFLD). As a result, many pathways relevant to steatosis were identified at the later time points (8 and 24 h) without a strong impact of the dose (Figure 3).

Thus, our pathway enrichment analysis supports the differentiation between steatotic and non-steatotic chemicals. This finding aligns with previous studies on other hepatotoxicants with diverse modes of action utilizing the TG-GATEs database. For example, de-regulation of selected modules in PHHs was specifically observed upon exposure to hepatocarcinogens [35]. However, the corresponding gene expression signatures were not conserved in primary rat hepatocytes (PRHs) or rat livers, suggesting species-specific differences in bioactivation, metabolism and mode of action. In contrast, for acutely cytotoxic compounds, the induction of a limited number of stress-response genes was conserved between PHHs and PRHs, thereby predicting hepatotoxicity in rats [36]. Similarly, gene expression analysis via TempO-Seq technology in human-induced pluripotent stem cell-derived hepatocyte-like cells, revealed modules related to endoplasmic reticulum (ER) stress and the unfolded protein response (UPR) that were specifically triggered by acutely cytotoxic hepatotoxicants [37].

### 2.3. Enrichment Analysis Based on mRNA Levels De-Regulated upon Exposure to Steatotic Chemicals Reveals Several KEGG Pathways Related to Steatosis

Pathways with particular relevance for steatosis, such as the MIEs PPAR and mTOR signalling pathways as well as KEs such as cholesterol metabolism and chemical carcinogenesis-reactive oxygen species were chosen for further investigation. The Kyoto Encyclopedia of Genes and Genomes (KEGG) web tools were used to depict relevant pathways.

**Figure 4:**
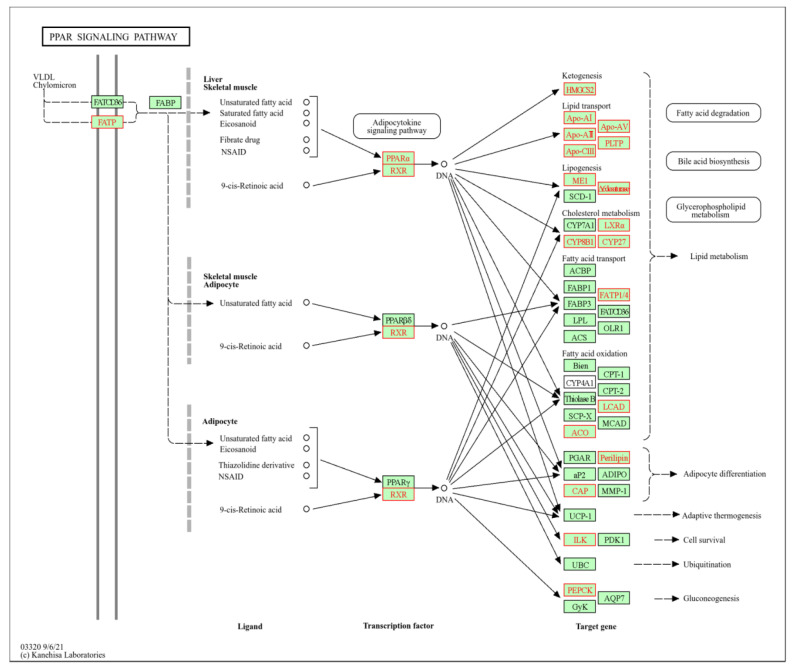
KEGG pathway analysis and DEVEA reveals PPAR signalling as a target of steatotic chemicals. In total, 3 analysis groups (doses: low, medium and high and all time points pooled) were used to identify pathways based on the comparison of DEGs induced by steatotic compounds relative to untreated cells and cells treated with non-steatotic compounds. Red boxes indicate up-regulated genes.

The full PPAR signalling pathway is presented in Figure 4 as provided by the KEGG database, with genes colour-coded to indicate their regulation derived from the DEVEA of the dose-dependent differences in treated cells versus pooled controls (T/PC, adj. *p*-value (0.05). Activation of PPARs is recognized as a key MIE in steatosis according to the current AOPs ([27,28]; Supplemental Figure S4).

As depicted in Figure 4, genes related to lipid transport (APOA4, APOA5), cholesterol metabolism (CYP27A1, CYP8B1), fatty acid oxidation (LCAD, ACO), and in general to lipid homeostasis such as fatty acid transporter (FATP1/4) were found to be specifically up-regulated. Notably, PPAR-alpha (PPARA) and its heterodimerization partner retinoid X receptor alpha (RXRA) were also induced.

The peroxisome proliferator-activated receptor (PPAR) family of nuclear hormone receptors plays a crucial role in regulating hepatic lipid metabolism and inflammation, both of which are central to the pathogenesis of steatosis and MASLD. There are three subtypes of PPARs (α, β/δ and γ), each with distinct roles and expression patterns. In hepatocytes, PPAR α plays a role in fatty acid uptake and ß-oxidation as well as bile and amino acid metabolism. In contrast, PPARß/δ is mainly involved in de novo lipogenesis, glucose utilization, and also ß-oxidation. PPAR γ is critical for fatty acid uptake and lipid droplet formation, which leads to elevated fat content of hepatocytes in the context of steatosis [38,39,40,41]. Hence, the observed deregulation of PPAR signalling by steatotic chemicals is in line with previous observations [27,42,43]. Whereas the activation of PPAR ß/δ and PPAR γ typically elevates lipid content, PPAR α signalling generally reduces intracellular lipid accumulation. That steatotic chemicals regulate the PPAR α pathway might seem counterintuitive, yet similar findings have been reported in liver transcriptome analysis from mice exposed to amiodarone and valproic acid [44]. Such up-regulation of PPAR α signalling may reflect an adaptive response aimed at countering detrimental excessive lipid accumulation. Based on the gene expression data, both positive and negative regulators of lipid accumulation appear to be affected. As discussed above, genes involved in fatty acid oxidation (LCAD, ACO) and PPARα signalling are generally associated with reduced lipid content, whereas genes involved in lipid (APOA4, APOA5) and fatty acid transport (FATP1/4) are linked to increased lipid accumulation. These findings illustrate the complexity of transcriptomic responses and highlight the limitations of such approaches when attempting to draw direct conclusions at the functional level. Consequently, transcriptomics should primarily be used to generate hypotheses that require validation through biochemical follow-up experiments, such as the measurement of intracellular lipid content in this case.

In addition to an impact on PPAR signalling and lipid homeostasis, cholesterol metabolism was affected upon treatment with steatotic chemicals (Supplemental Figures S9 and S10). In accordance with an impact on lipid homeostasis, several mRNAs encoding enzymes involved in glycerophospholipid metabolism and fatty acid degradation were affected upon treatment with steatotic chemicals (Supplemental Figures S11–S14). Also, the KEGG pathway of chemical carcinogenesis/reactive oxygen species was impacted by steatotic chemicals (Supplemental Figure S15). Moreover, the mRNAs of genes implicated in the mTOR signalling pathway were induced (Supplemental Figure S16), including genes related to MAPK signalling (Ras, MEK, ERK), autophagy, mitophagy, and glycerophospholipid metabolism (S6K1, Lipin-1, MLST8).

Beyond the analysis of the transcriptome data by DEVEA of treated cells versus pooled controls, GSEA also indicated an impact on cholesterol metabolism (Supplemental Figures S17 and S18). Highlighting effects on cholesterol transport and high-density lipoproteins by treatment with steatotic chemicals.

**Figure 5:**
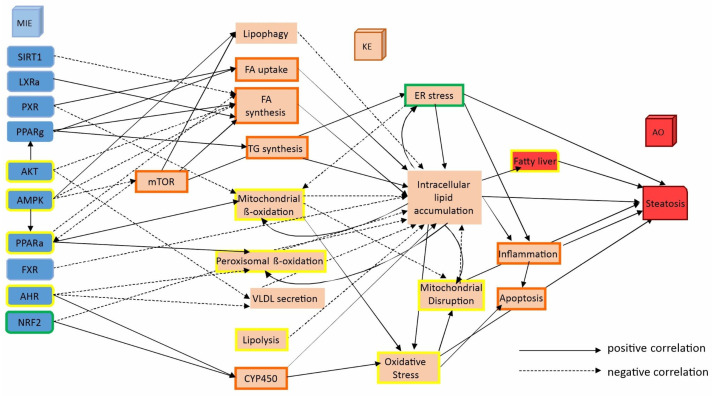
Mapping of identified pathways interrogating the primary hepatocyte microarray datasets onto the established adverse outcome pathway for chemical-induced liver steatosis. Green borders indicate modules derived solely from GSEA; yellow borders indicate modules derived solely from DEVEA analysis; orange borders indicate modules derived from both analysis methods. The solid lines represent a positive correlation between the upstream and downstream KEs, while the dashed lines indicate a negative correlation. Abbreviations: AHR, aryl hydrocarbon receptor; AKT, serine-threonine protein kinase; AMPK, adenosine monophosphate-activated protein kinase; CYP450, cytochrome P450; ER, endoplasmic reticulum; FA, fatty acid; LXRα, liver X receptor alpha; mTOR, mechanistic target of rapamycin kinase; NRF2, nuclear factor erythroid 2-related factor 2; PPARα, peroxisome proliferator-activated receptor alpha; PPARγ, peroxisome proliferator-activated receptor gamma; PXR, pregnane X receptor; SIRT1, sirtuin 1; TG, triglyceride; VLDL, very-low-density lipoprotein. Note that some KEs, such as, e.g., oxidative stress and inflammation, are not unique to steatosis but are also involved in other AOPs.

In summary, our transcriptome analysis employing GSEA or DEVEA revealed critical pathways known to regulate steatosis which could be mapped onto the established AOP (Figure 5). Enriched pathways of steatotic chemicals include those involved in fatty acid uptake, fatty acid homeostasis, cholesterol metabolism and mTOR signalling [10,27,45,46,47]. As already discussed above, dependent on the database and bioinformatic tools (i.e., GSEA and DEVEA) overlapping and complementary pathways were identified, resulting in a broader coverage of relevant events related to the AOP of steatosis. However, our transcriptomic approach could not fully recapitulate the entire AOP, which is not unexpected. The AOP framework was established through an AI-assisted literature search that extracted information from 176 publications encompassing different species, various test systems (both in vitro and in vivo), and a much broader range of steatotic chemicals. Nevertheless, our more limited analysis in primary hepatocytes identified de-regulated genes that are associated with several key events of the established AOP. These findings demonstrate the usefulness of TG-GATEs data and our subsequent analytical approaches for the categorization of steatotic chemicals.

To further explore biological processes de-regulated by steatotic chemicals, we employed a knowledge graph and webserver application, Enrichr-KG [48]. Enrichr-KG enables the visual representation of associations between genes and functional terms, such as gene ontology (GO) terms or mammalian phenotypes, to allow data integration across multiple datasets. Based on the comparison of DEGs altered by steatotic compounds relative to untreated cells and cells treated with non-steatotic compounds as identified by DEVEA, we performed Enrichr-KG analysis considering dose and time.

**Figure 6:**
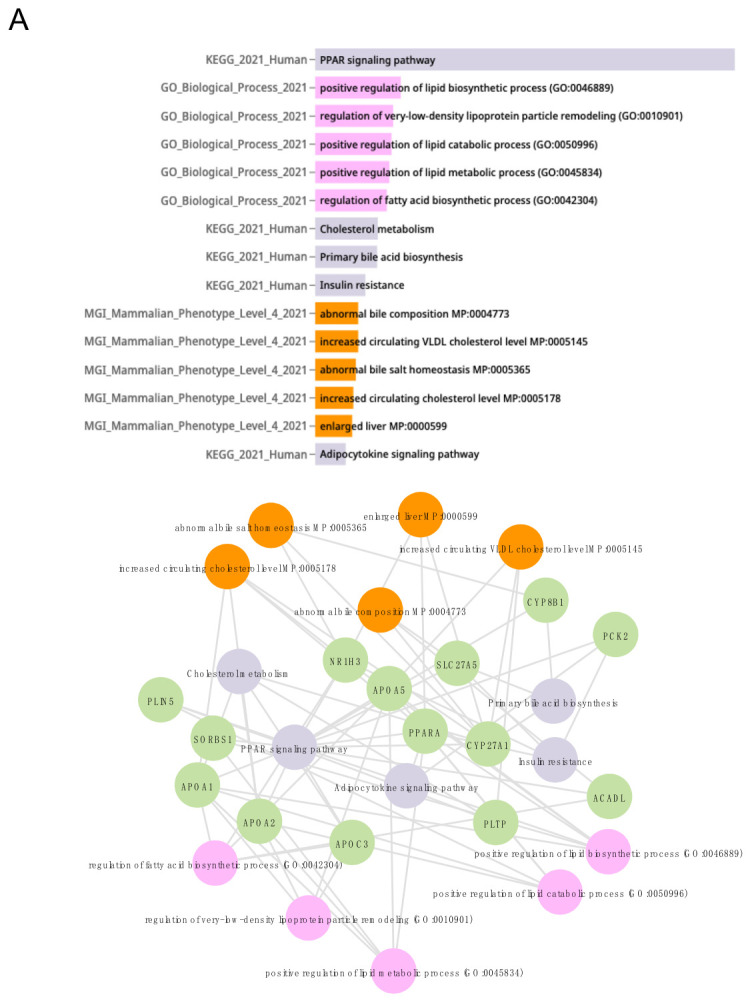

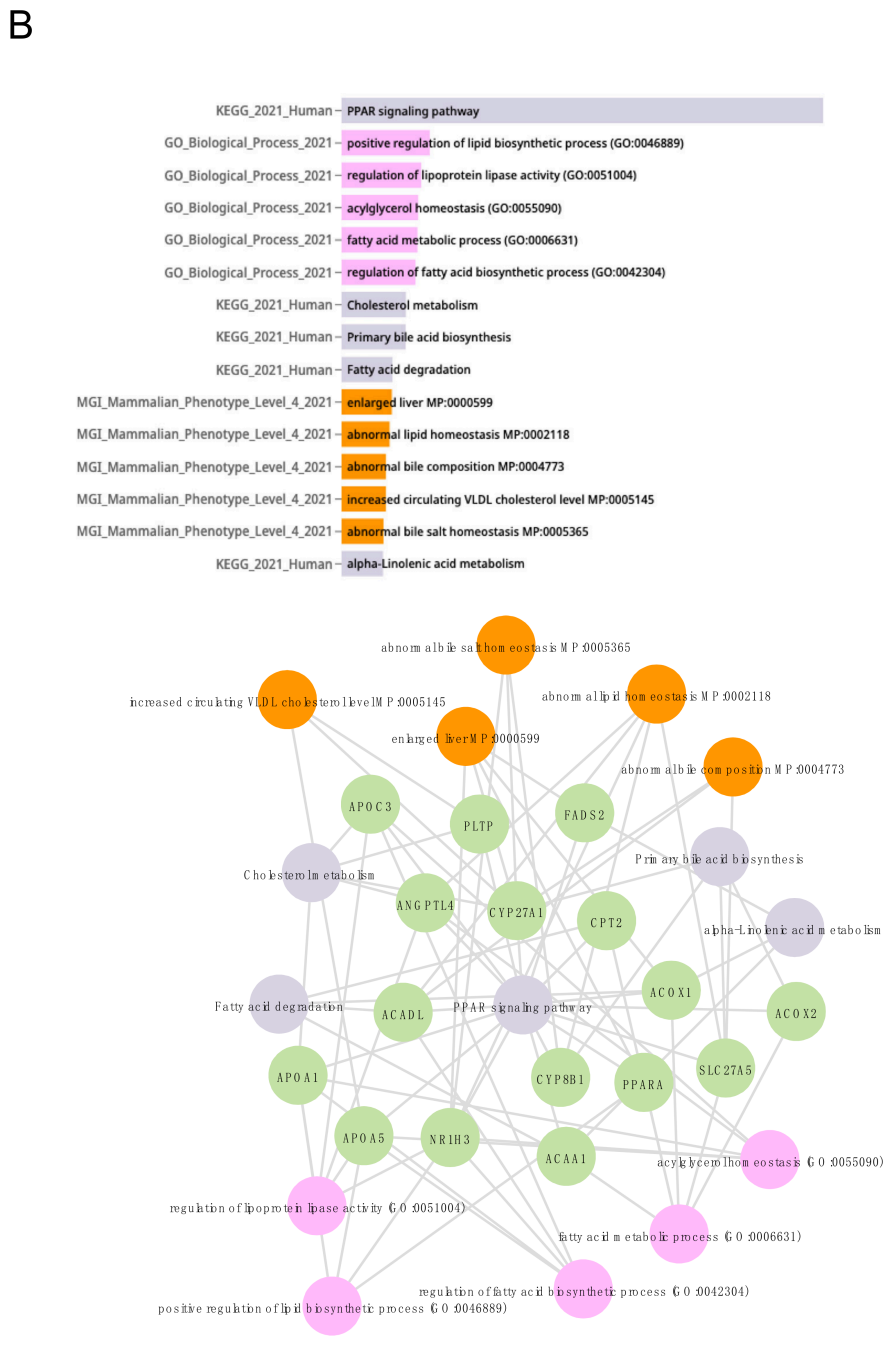
Enrichr-KG analysis and DEVEA links gene sets de-regulated by steatotic chemicals to PPAR signalling, associated biological processes and adverse mammalian phenotypes. (**A**) Dose-dependent analysis. In total, 3 analysis groups (doses: low, medium and high and all time points pooled) were used to identify DEGs induced by steatotic compounds relative to untreated cells and cells treated with non-steatotic compounds. Bar chart of enriched terms ordered by *p*-value and network view. Colour code: grey—KEGG terms, pink—GO terms, orange—mammalian phenotypes, and green—de-regulated genes. (**B**) Time-dependent analysis (exposure time: 2, 8 and 24 h and all doses pooled).

The functional enrichment analysis conducted with Enrichr-KG also revealed PPAR signalling as a critical pathway (Figure 6). In addition to PPAR signalling, KEGG terms related to insulin resistance, cholesterol metabolism, primary bile acid synthesis, and fat digestion and absorption were also significantly affected. GO terms enriched in the dataset encompassed processes linked to lipid biosynthesis and metabolism as well as bile acid metabolism. Furthermore, mammalian phenotype ontology associations link the gene set de-regulated by steatotic chemicals to known key events in steatosis, i.e., increased circulating cholesterol and abnormal lipid homeostasis.

When separately considering the dose or time for the analysis, some differences could be observed. For example, a dose-dependent link to lipid catabolic processes (Figure 6A, left panel) was found, while a time-dependent impact on acylglycerol homeostasis could be detected (Figure 6B, left panel). Interestingly, the individual analysis based on dose and time indicates an adverse outcome at the organ level; that is, an enlarged liver and abnormal bile salt homeostasis. These phenotypes are recognized as early indicators of steatosis and potential liver inflammation [39,42,43,49].

Additional Enrichr-KG results connected the de-regulated gene set to cholesterol metabolism, with GO and KEGG terms as well as phenotypes pertinent to transport, homeostasis and efflux of cholesterol and sterol for both analysis methods, i.e., DEVEA and GSEA (Supplemental Figures S19 and S20), found to be linked. However, Enrichr-KG results based on DEVEA and the comparison of treated cells versus untreated cells and samples of cells treated with non-steatotic compounds show a broader coverage of pathways than those found using GSEA, as an impact on mTOR and ROS could also be uncovered (Supplemental Figures S21 and S22).

In essence, Enrichr-KG analysis provides additional insights into de-regulated pathways but also mammalian phenotypes and adverse outcomes, which were not highlighted by the previous analysis tools.

### 2.4. Metabolomics Can Distinguish Between Steatotic and Non-Steatotic Chemicals

**Figure 7:**
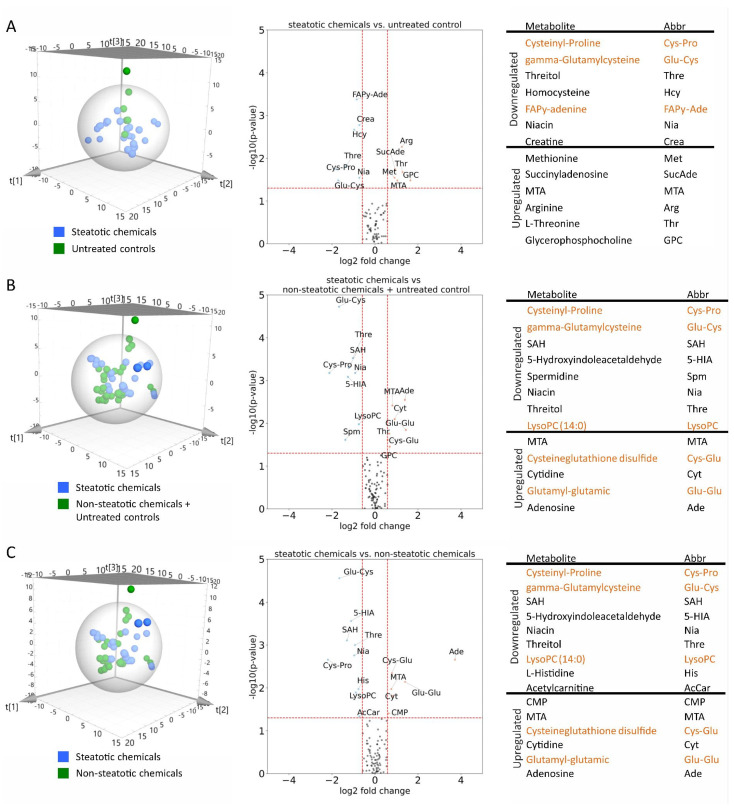
Metabolomics can distinguish steatotic from non-steatotic chemicals. Human liver cells (HepG2) were treated for 24 h with four different concentrations (1, 10, 100 and 1000 μM) of steatotic and non-steatotic chemicals and the metabolites were analyzed. (**A**–**C**) Unsupervised PCA (left panels): Cells treated with steatotic compounds (blue) were compared to (**A**) unexposed controls (green), (**B**) unexposed controls and cells treated with non-steatotic compounds, and (**C**) cells treated with non-steatotic chemicals. Right panels and tables list the de-regulated metabolites (labelled in orange are selected metabolites related to oxidative stress). Abbreviations: MTA: Methylthioadenosine, SAH: S-Adenosylhomocysteine, FAPγ: 4,6-Diamino-5-(formylamino)pyrimidine, CMP: L-L-Homoglutathione, LysoPC: Lysophosphatidylcholine.

In addition to transcriptomics, we interrogated metabolomics data derived from a previous study on human HepG2 cells, which have been exposed for 24 h to an overlapping dose range [50] to distinguish steatotic and non-steatotic chemicals through biological activity profiling (for the list of compounds and concentrations, see Supplemental Table S1C). Unsupervised PCA showed different clusters for all three comparisons: unexposed controls versus cells treated with steatotic chemicals (Figure 7A), cells exposed to steatotic chemicals compared to those treated with non-steatotic chemicals combined with the untreated controls (Figure 7B) or cells exposed to steatotic chemicals compared to those treated solely with non-steatotic chemicals (Figure 7C). To identify metabolites associated with steatosis, we performed supervised PLS-DA and obtained robust models for the comparisons involving steatotic chemicals versus either non-steatotic chemicals alone or in combination with the unexposed controls (Supplemental Figure S23B,C), while only a weak model was obtained for the comparison between steatotic and control samples (Supplemental Figure S23A). This indicates that metabolic alterations induced by steatotic compounds are more distinctive when contrasted with non-steatotic exposures than with unexposed controls, suggesting a specific metabolic signature associated with steatosis rather than general exposure effects. The significant metabolites obtained from these models were further filtered by univariate analysis and visualized in volcano plots (Figure 7). These included amino acids, lipids, and intermediates involved in glutathione and cysteine metabolism. Similar metabolomic changes have been previously associated with metabolic dysfunction-associated steatotic liver disease (MASLD) [51].

Specifically, changes in the levels of gamma-glutamylcysteine (Glu-Cys), cysteine glutathione disulphide (Cys-Glu), and glutamyl-glutamic (Glu-Glu) were noteworthy. Cys-Glu is an oxidation product of glutathione, whereas Glu-Cys serves as a precursor, and Glu-Glu is a metabolite of glutathione. These findings suggest that steatotic chemicals disrupt glutathione homeostasis, indicating oxidative stress [50,52,53]. Although a direct comparison between the metabolomic data obtained in HepG2 cells and the transcriptomic changes observed in primary hepatocytes was not performed—due to differences in cell types, exposure times, and concentrations—oxidative stress-related processes were nevertheless identified as being affected in the transcriptomic analysis described above. This disruption is consistent with alterations in lysophosphatidylcholine (LysoPC), a known marker of oxidative damage to membrane phospholipids [54].

Hence, in addition to transcriptome analysis of exposed primary human hepatocytes, metabolomic data obtained from HepG2 cells provide a complementary means to separate steatotic from non-steatotic chemicals, confirming a specific impact of steatosis on the metabolome. Given the differences in the two cell models, future studies should combine different omics modalities in the same model, preferentially in primary cells.

A primary limitation of our study, as well as similar approaches, is the inherent nature of assessing steatosis in vitro over relatively short exposure periods (up to 24 h). Such short time frames are well-suited for identifying molecular initiating events (MIEs), which represent the initial interaction of a chemical with a molecular target and the resulting early transcriptomic responses. However, short-term in vitro models cannot capture the long-term physiological accumulation of lipids or the ultimate adverse outcome, which typically develops over extended biological time scales. Furthermore, although primary human hepatocytes (PHHs) represent a highly relevant model for the identification of hepatic MIEs, they lack the systemic and multi-organ interactions, such as obesity and insulin resistance, that drive the multifactorial nature of metabolic dysfunction-associated steatotic liver disease (MASLD) in vivo. Despite these limitations, the identification of early predictive markers remains crucial for grouping chemicals and assessing their potential to induce steatosis, while reducing reliance on long-term animal studies.

## 3. Materials and Methods

### 3.1. Chemical Selection

Thirty-one steatotic and nine non-steatotic compounds were selected based on the literature and available data. In the next step, microarray datasets could be found for four of the selected steatotic compounds (amiodarone, carbon tetrachloride, tamoxifen and valproic acid) and for two non-steatotic chemicals (colchicine and imipramine). Metabolite data were available for six steatotic (amiodarone, cyclosporine A, fialuridine, tamoxifen, tetracycline, and valproic acid) and six non-steatotic compounds (amikacin, citrate, colchicine, cumene hydroperoxide, gentamicin, and imipramine). All compounds and concentrations are listed in Supplemental Table S1.

### 3.2. Microarray Dataset

Microarray data were obtained from Open TG-GATEs [55]. The accession TG Gates key is E-MTAB-798. In this data a total of 108 in vitro samples of primary human hepatocytes were exposed to a variety of chemicals at three dose levels: low, middle, and high. The highest dose was defined as the concentration causing a 20% release of lactate dehydrogenase, while the middle and low doses were set to one-fifth and one-twenty-fifth of that concentration, respectively. Exposure times were 2, 8, or 24 h with two biological replicates each and GeneChip analysis was performed in duplicate for each concentration using U133 Plus 2.0 arrays for human hepatocytes (Affymetrix, Santa Clara, CA, USA) [56,57].

Raw CEL files were processed in R using the expresso function (from the affy package). The pipeline comprised RMA background correction (bgcorrect.method = “rma”), a constant normalization approach (normalize.method = “constant”), PM-only probe correction (pmcorrect.method = “pmonly”), and a median polish summarization (summary.method = “medianpolish”). To ensure the most up-to-date gene annotations, a custom BrainArray CDF (version 25) was employed. The resulting normalized expression matrices were then used for subsequent analyses.

### 3.3. Metabolomics Dataset

The data was sourced from Martínez-Sena et al. [50], selecting specific steatotic and non-steatotic chemicals to match transcriptomic data (see Section 3.1). As described in Martínez-Sena et al., for this dataset human liver cells (HepG2) were treated for 24 h with four different concentrations of drugs (1, 10, 100 and 1000 μM), or up to maximum solubility, and the metabolites (116 features total) were analyzed. The data were autoscaled and mean-centred.

### 3.4. Control Groups and Parameters Used for DEG Analysis

Samples treated with steatotic compounds were compared to unexposed controls. In addition to the classical approach, samples exposed to steatotic compounds were also compared to a pool of unexposed controls and samples treated with non-steatotic compounds. The latter method should assess whether the comparison of steatotic versus non-steatotic chemicals can further improve the categorisation of steatotic chemicals.

Analysis was conducted for dose and time, using either the unexposed or pooled control. For the time analysis, data were split by time points, including all doses for each time point. For the dose analysis, groups were defined by low, medium, and high doses, each including all time points.

### 3.5. Identification of Differentially Expressed Genes (DEGs)

The Omics Data Analysis Framework for Regulatory Application (R-ODAF) was employed with the following criteria applied in the analysis as cut-off parameters: adjusted *p*-value 0.05 (false discovery rate, FDR) and average expression ≥ 6. No logFC threshold was set, to enable detection of small but significant changes. All analyses were carried out using the parameters and group of samples as described in Section 3.4. As low concentrations of chemicals were used, this approach often resulted in very low numbers of DEGs for many analysis groups. For those cases, a different statistical approach was used based on the ranking by the *t*-statistic value and a *p*-value of 0.05 as described in Section 3.6 [58]. While the baseline GeneChip analysis for individual concentrations was performed in duplicate, the statistical power of our study does not rely on single-compound replicates. Instead, our methodological novelty lies in a broad pooling strategy designed to overcome compound-specific noise. We evaluated 4 steatotic and 2 non-steatotic compounds across multiple doses and time points. By grouping multiple chemicals according to their mode of action and comparing them to a pooled control group (comprising untreated cells and cells treated with non-steatotic compounds), we vastly increased the effective sample size. This approach successfully amplified the relative differences in gene expression tied to the underlying process of steatosis rather than isolated chemical noise.

### 3.6. Functional and Pathway Enrichment Analysis

Gene set enrichment analysis (GSEA, Broad Institute Inc., Massachusetts Institute of Technology, Cambridge, MA, USA, Version 4.4.0) was performed with a pre-ranking by t-statistic. The enrichment was run with gene set database h.all.v2024.1.Hs.Symbols.gmt with 1000 permutations, weighted enrichment statistics and meandiv normalization. A cut-off for the adjusted-*p*-value (FDR) of 0.05 was used to select DEGs for further processing employing the databases GO biological processes, hallmark genes and WikiPathways [59,60,61,62]. All data passing the filter of an adjusted-*p*-value of 0.05 (as described in Section 3.5) were analyzed further by differential expression analysis and the data visualization and enrichment analysis of transcriptomics data (DEVEA) tool [63]. The Kyoto Encyclopedia of Genes and Genomes (KEGG) database was employed for further pathway enrichment analysis. Gene ontology (GO) analysis was carried out with Enrichr-KG [48,64,65,66,67,68,69,70]. The time and dose-dependent analysis of DEGs and subsequent pathway enrichment analysis was performed for the three time points (2, 8 and 24 h) and the three doses (low, medium and high), resulting in 9 analysis groups. A pathway was followed up further if detected at least in 2 out of the 9 analysis groups. DEGs were included in the representative pathway maps if detected at least in 2 out of the 9 analysis groups. Heatmaps were created with the R-package ggplot2. All identified KEGG pathways are listed for the dose- and time-dependent analysis in Tables S2 and S3, including the number and names of the affected genes for each pathway.

### 3.7. Metabolomics

Multivariate models were generated with the software SIMCA© 17(Sartorius). Unsupervised Principal Component Analysis (PCA) was applied to visualize clustering trends. Partial Least Squares Discriminant Analysis (PLS-DA) modelling was performed to identify the significantly altered metabolites with the obtained Variable Importance in Projection (VIP) values. The significance of metabolites with VIP value < 1 was further confirmed by univariate statistical analysis between the groups (Student’s t-test for variables with a normal distribution and Mann–Whitney U test for the rest) and visualized through volcano plots using in-house Python 3.7.11 scripts employing the Matplotlib 3.5.3 library [71,72,73].

## 4. Conclusions

By employing microarray and metabolomics datasets obtained from exposure of human hepatocytes, we successfully distinguished the biological activities of steatotic and non-steatotic chemicals. Pathway analysis revealed important molecular initiating events, key events and adverse outcomes relevant for steatosis. Specifically, genes involved in lipid transport, cholesterol metabolism, fatty acid oxidation, lipid homeostasis such as fatty acid transport, oxidative stress and PPAR-alpha signalling were found to be up-regulated. Considering the relative transcript or metabolite levels which were in common upon treatment with steatotic chemicals, two different comparisons could be made. Relative changes were either normalized to the relative levels recorded in untreated control cells or to the average levels measured in untreated cells and cells treated with non-steatotic compounds. The pooled control approach yielded a clearer distinction of steatotic chemicals, as evidenced by a greater number of differentially expressed genes and pathways with specific relevance to steatosis. However, for only two non-steatotic chemicals were microarray data from TG-GATE available, which is clearly a limitation of this and similar studies. Once more datasets become available, including RNA sequencing data, larger group sizes could be investigated. For the most comprehensive coverage of an AOP it seems helpful to combine the different strategies, considering also time- and dose-dependent changes, and to employ different databases. In perspective, this strategy could be applied to other classes of chemicals targeting different adverse outcome pathways, potentially improving chemical grouping based on transcriptome and metabolome profiling. Furthermore, integrating additional unbiased in silico approaches, including machine learning and artificial intelligence, might further support next-generation risk assessment procedures. This strategy also exemplifies how systems toxicology can contribute to a next-generation risk assessment framework.

## Data Availability

The supporting data for the DEG study is available on the BioStudies repository (http://www.ebi.ac.uk/biostudies; URL accessed on 14 April 2026) accession number E-MTAB-798. The supporting data of the Metabolomics study are available on the Zenodo repository (https://zenodo.org/) accession number 6411855.

## Supplementary Materials

The following supporting information can be downloaded at https://www.mdpi.com/article/10.3390/ijms27093825/s1.

## Abbreviations

The following abbreviations are used in this manuscript:

ACO	Acyl-CoA oxidase 1
AMPK	Adenosine monophosphate-activated protein kinase
AOP	Adverse outcome pathway
APOA4	Apolipoprotein A4
APOA5	Apolipoprotein A5
ASPIS	Animal-free safety assessment of chemicals: project cluster for implementation of novel strategies
ATG1	Serine/threonine-protein kinase ULK1
CYP27A1	Cholestanetriol 26-monooxygenase
CYP8B1	Sterol 12-alpha-hydroxylase
Cys-Glu	Cysteine glutathione disulphide
DEVEA	Differential expression analysis
ERK	Mitogen-activated protein kinase 1/3
FATP1/4	Solute carrier family 27 (fatty acid transporter)
Glu-Cys	Gamma-glutamylcysteine
Glu-Glu	Glutamyl-glutamic
GO	Gene ontology
KE	Key event
KEGG	Kyoto encyclopedia of genes and genomes
LCAD	Long-chain-acyl-CoAdehydrogenase
Lipin-1	Phosphatidatephosphatase LPIN
LysoPC	Lysophosphatidylcholine
MASLD	Metabolic dysfunction-associated steatotic liver disease
MEK	Mitogen-activated protein kinase kinase1
MIE	Molecular initiating event
MLST8	Target of rapamycin complex subunit LST8
mTOR	Mammalian target of rapamycin
NAFLD	Non-alcoholic fatty liver disease
NAM	New approach methodology
OECD	Organisation for economic co-operation and development
OORF	OECD omics reporting framework
PCA	Principal component analysis
PEA	Pathway enrichment analysis
PHH	Primary human hepatocyte
PLS-DA	Partial least squares discriminant analysis
PPAR	Peroxisome proliferator-activated receptor
PRH	Primary rat hepatocyte
Ras	GTPase HRas
R-ODAF	Omics data analysis framework for regulatory application
RXRA	Retinoid X receptor alpha
S6K1	Ribosomal protein S6 kinase beta
TG-GATEs	Toxicogenomics project-genomics assisted toxicity evaluation system
TNF	Tumour necrosis factor
UPR	Unfolded protein response
