# Supplementary material for: Discrimination of Steatotic and Non-Steatotic Chemicals Through Transcriptome Analysis in Primary Human Hepatocytes
Source: Int J Mol Sci. 2026 Apr 25;27(9):3825. doi: 10.3390/ijms27093825 (PMC13164476; doi:10.3390/ijms27093825)
Supplement: Supplementary file 1 [file ijms-27-03825-s001.zip › ijms-4139508-supplementary120426.pdf]

Supplementary information to:

Discrimination of Steatotic and Non-Steatotic Chemicals Through Transcriptome Analysis in Primary Human Hepatocytes

Christina A. Cramer von Clausbruch [1], Marcha Verheijen [2], Giulia Callegaro [3], Jonathan H. Freedman [4], Rita Ortega-Vallbona [5], Martina Palomino-Schätzlein [5,6], Florian Caiment [2] and Carsten Weiss[1*]

[1] Karlsruhe Institute of Technology (KIT), Institute of Biological and Chemical Systems, Biological Information Processing, 76344 Eggenstein-Leopoldshafen, Germany;c.cramer-von-clausbruch@gmx.de (C.A.C.v.C.);carsten.weiss@kit.edu (C.W.)

[2] Maastricht University,Department of Translational Genomics - Research Institute for Oncology and Reproduction, 6229 Maastricht, The Netherlands; verheijen.m@gmail.com (M.V.) ;florian.caiment@maastrichtuniversity.nl (F.C.)

[3] Division of Cell Systems and Drug Safety, Leiden Academic Centre for Drug Research, Leiden University, Einsteinweg 55, 2333 C C Leiden, The Netherlands; g.callegaro@lacdr.leidenuniv.nl (G.C.)

[4] University of Birmingham,Centre for Environmental Research and Justice and School of Biosciences,B15 2TT Birmingham,UK;jon.freedman@wormtox.org

[5] ProtoQSAR SL.,Parque Tecnológico de Valencia, 46980 Paterna, Spain; rortega@protoqsar.com (R.O.-V.),mpalomino@protoqsar.com (M.P.-S.)

[6] MolDrug AI Systems SL, 46018 Valencia, Spain.;mpalomino@protoqsar.com (M.P.-S.)

* Correspondence: carsten.weiss@kit.edu (C.W.)

A

Steatotic	Non-Steatotic
2-((2-Nitro-4-(trifluoromethyl)phenyl)amino)ethanol	Amikacin
2-(2,5-Diaminophenyl)ethanol sulfate	**Colchicine**
2-Aminophenol	Cumene hydroperoxide
2-ethylhexyl diphenyl phosphate	**Imipramine**
5-Amino-6-chloro-o-cresol	Minocycline
**Amiodarone**	Rotenone
Benzenamine	Trisodium citrate
**Carbon tetrachloride**	tert-Butyl hydroperoxide
Cetylpyridinium chloride	Tigecycline
Chloroform	
Clothianidin	
Cyproconazole	
Decamethylcyclopentasiloxane	
Imazalil	
Lilial	
N-(2,2,2-Trifluoroethyl)-N-{4-[2,2,2-trifluoro-1-hydroxy-1-ethyl]phenyl} benzenesulfonamide	
N,N'-Bis(2-hydroxyethyl) -2-nitro-p-phenylenediamine	
New fuchsine	
N-methyl pyrroliidone	
Perfluorooctanoic acid	
Phenoxyethanol	
**Tamoxifen**	
Tetrachloroethylene	
Tetracycline	
Thiacloprid	
Toluene	
Tributyltin	
Tributyltin fluoride	
Tricresyl phosphate (TMPP)	
**Valproic acid**	
Vinyl chloride	

B

	Low Dose	Middle Dose	High Dose
Amiodarone	0.28	1.4	7
Carbon tetrachloride	300	1500	7500
Tamoxifen	0	5	25
Valproic acid	200	1000	5000
Colchicine	0	800	4000
Imipramine	0	4	15

C

Steatotic	Non-Steatotic
Amiodarone	Amikacin
Cyclosporine A	Citrate
Fialuridine	Colchicine
Tamoxifen	Cumen hydroperoxide
Tetracycline	Gentamicin
Valproic acid	Imipramine

Table S1: A: List of selected 31 known steatotic compounds and 9 non-steatotic compounds. Compounds for which transcriptome and metabolome data are available are in bold. B: List of low, middle and high dose concentration in micromolar (µM) of transcriptomics data set. C: List of all compounds used in the metabolomics study. Compound treatment concentration was set to 1, 10, 100 and 1000 µM. Due to limited solubility, the following compounds were incubated at their highest possible concentration: Amiodarone (540 µM), Cyclosporine A (300 µM), Fialuridine (500 µM), and Tamoxifen (510 µM).

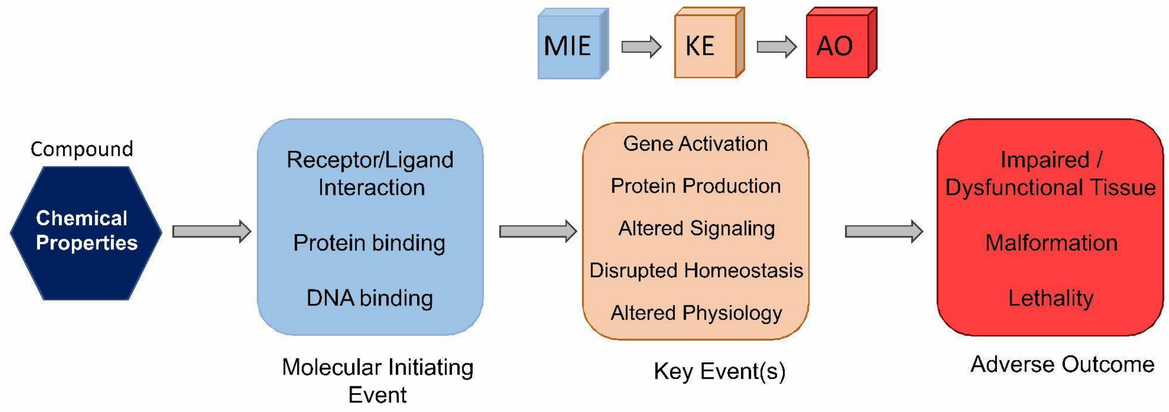

Supplemental Figure S1: A simplified view of an Adverse Outcome Pathway. Molecular Initiating Events (MIEs) lead to Key Events (KEs) which couple to Adverse Outcomes (AO).

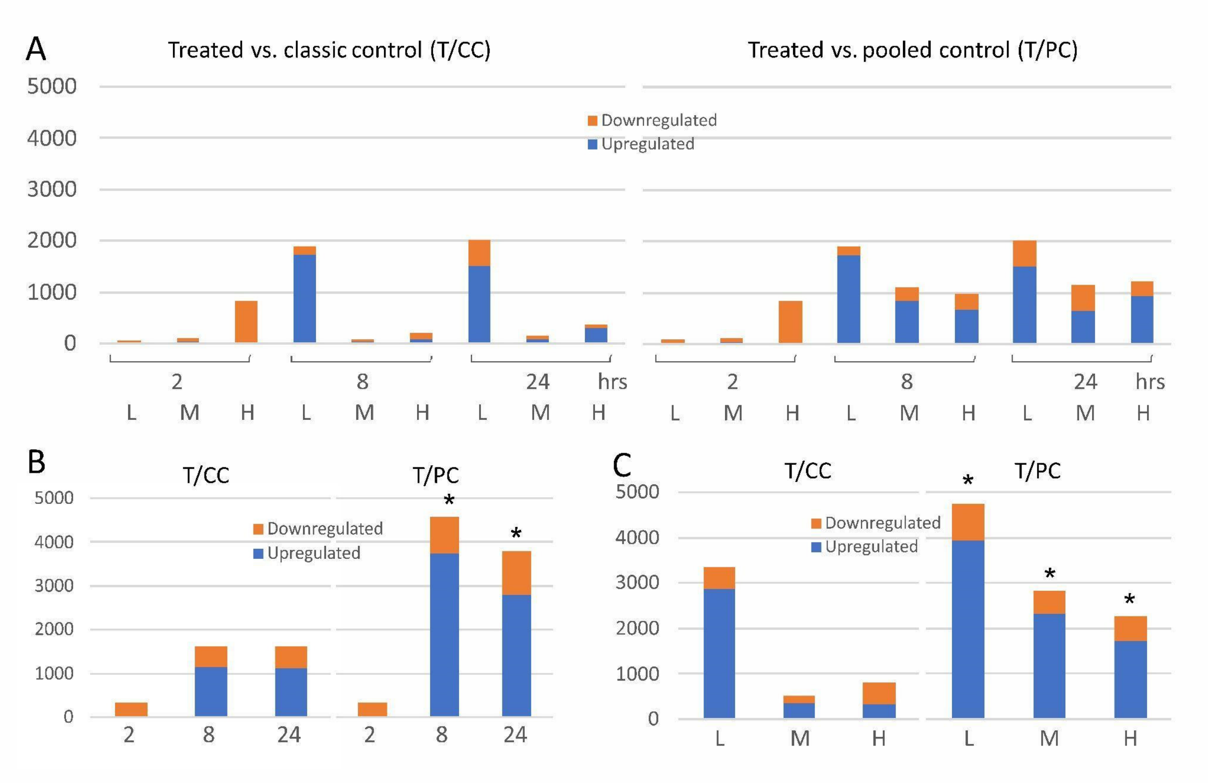

Supplemental Figure S2: The number of differentially expressed genes (DEG) induced by steatotic compounds is time - and dose-dependent. A: DEG analysis in primary human hepatocytes. Shown are the number of identified DEGs based on a logFC approach at different timepoints and doses. The relative transcript levels detected in cells upon treatment with steatotic chemicals were either normalized to the relative levels recorded in untreated control cells (left panel, treated vs. classic control: T/CC) or to the average levels measured in untreated cells and cells treated with non-steatotic compounds (right panel, treated vs. pooled control: T/PC). B: DEG analysis in dependence of time. All samples treated with the three doses were pooled and analysed to depict the impact of time. C: DEG analysis in dependence of dose. All samples treated at the three time points were pooled and analysed to depict the impact of dose. All DEGs which were derived based on an adjusted-p-value of 0.05 are marked with *, all other DEGs were derived based on a p-value of 0.05. (L=low dose, M=middle dose, H=high dose).

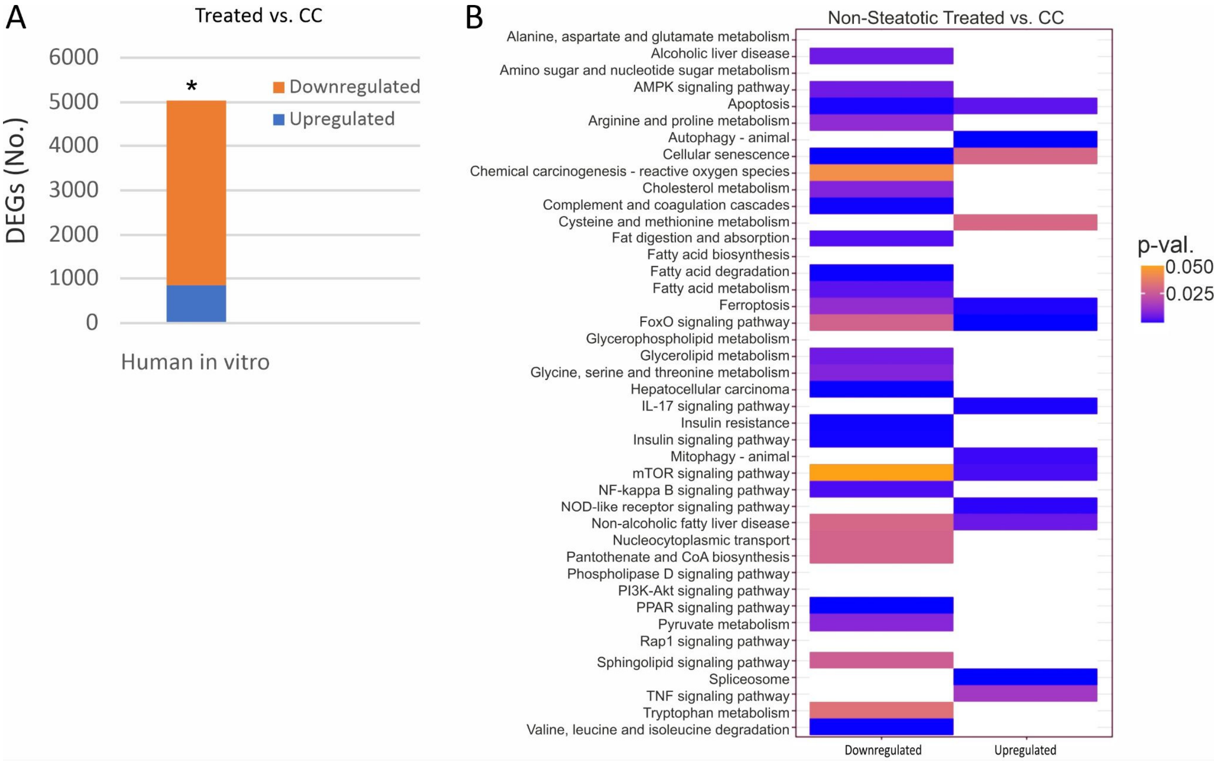

Supplemental Figure S3: Non-steatotic compounds mostly downregulate genes and pathways related to steatosis. A: The number of differentially expressed genes (DEG) induced by non-steatotic compounds compared to untreated controls (* indicates DEGs derived by adjusted p-value of 0.05). The relative transcript levels detected in human primary hepatocytes upon treatment with non-steatotic chemicals were normalized to the relative levels recorded in untreated control cells. B: Pathway Enrichment Analysis conducted for non-steatotic compounds compared to unexposed controls. Identified pathways are shown based on the comparison of DEGs induced by non-steatotic compounds relative to untreated cells.

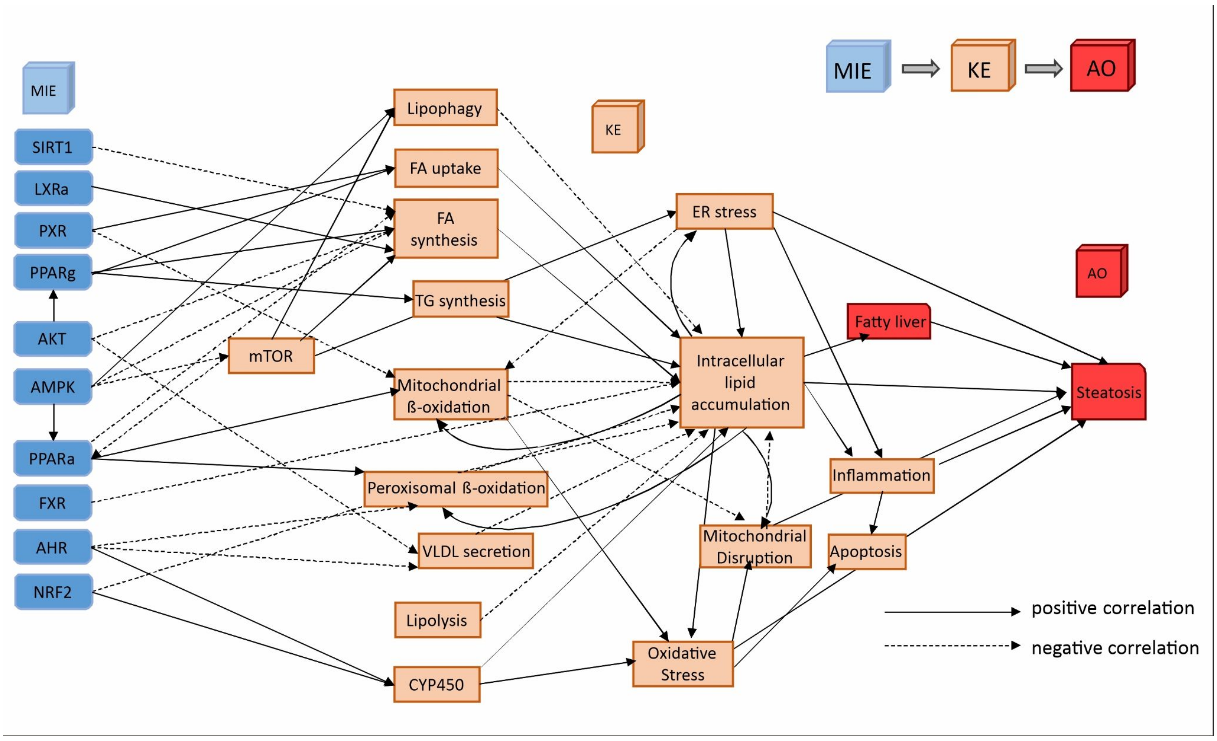

Supplemental Figure S4: Schematic overview of the most recent Adverse Outcome Pathway for chemical – induced liver steatosis. The solid lines represent a positive correlation between the upstream and downstream KEs, while the dashed lines indicate a negative correlation. Abbreviations: AHR, aryl hydrocarbon receptor; AKT, serine-threonine protein kinase; AMPK, adenosine monophosphate-activated protein kinase; CYP450, cytochrome P450; ER, endoplasmic reticulum; FA, fatty acid; LXRα, liver X receptor alpha; mTOR, mechanistic target of rapamycin; NRF2, nuclear factor erythroid 2-related factor 2; PPARα, peroxisome proliferator-activated receptor alpha; PPARγ, peroxisome proliferator-activated receptor gamma; PXR, pregnane X receptor; SIRT1, sirtuin 1; TG, triglyceride; VLDL, very-low-density lipoprotein.

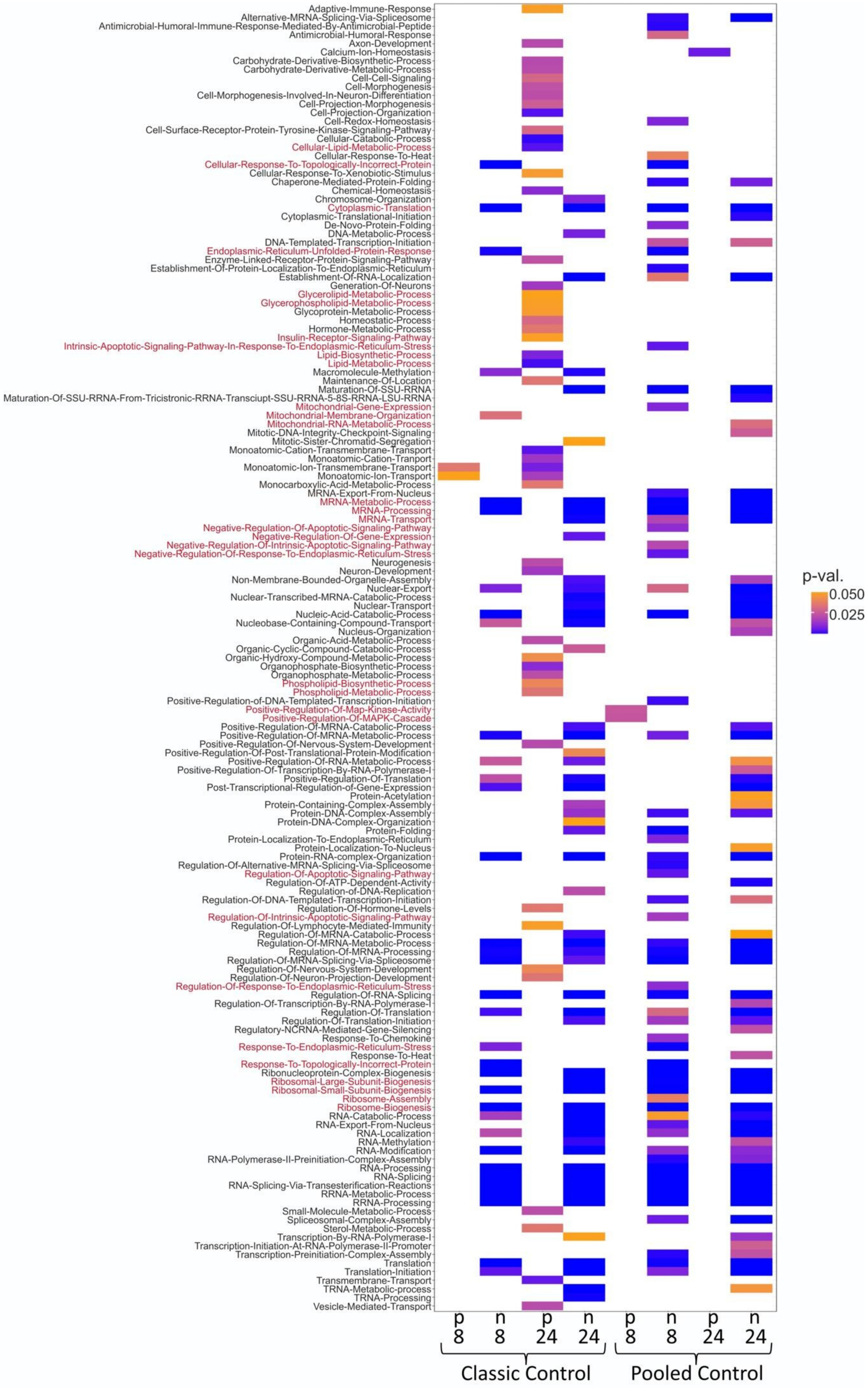

Adaptive-Immune-Response
Alternative-MRNA-Splicing-Via-Spliceosome
Antimicrobial-Humoral-Immune-Response-Mediated-By-Antimicrobial-Peptide
Antimicrobial-Humoral-Response
Axon-Development
Calcium-Ion-Homeostasis
Carbohydrate-Derivative-Biosynthetic-Process
Carbohydrate-Derivative-Metabolic-Process
Cell-Cell-Signaling
Cell-Morphogenesis
Cell-Morphogenesis-Involved-In-Neuron-Differentiation
Cell-Projection-Morphogenesis
Cell-Projection-Organization
Cell-Redox-Homeostasis
Cell-Surface-Receptor-Protein-Tyrosine-Kinase-Signaling-Pathway
Cellular-Catabolic-Process
Cellular-Lipid-Metabolic-Process
Cellular-Response-To-Heat
Cellular-Response-To-Topologically-Incorrect-Protein
Cellular-Response-To-Xenobiotic-Stimulus
Chaperone-Mediated-Protein-Folding
Chemical-Homeostasis
Chromosome-Organization
Cytoplasmic-Translation
Cytoplasmic-Translational-Initiation
De-Novo-Protein-Folding
DNA-Metabolic-Process
DNA-Templated-Transcription-Initiation
Endoplasmic-Reticulum-Unfolded-Protein-Response
Enzyme-Linked-Receptor-Protein-Signaling-Pathway
Establishment-Of-Protein-Localization-To-Endoplasmic-Reticulum
Establishment-Of-RNA-Localization
Generation-Of-Neurons
Glycerolipid-Metabolic-Process
Glycerophospholipid-Metabolic-Process
Glycoprotein-Metabolic-Process
Homeostatic-Process
Hormone-Metabolic-Process
Insulin-Receptor-Signaling-Pathway
Intrinsic-Apoptotic-Signaling-Pathway-In-Response-To-Endoplasmic-Reticulum-Stress
Lipid-Biosynthetic-Process
Lipid-Metabolic-Process
Macromolecule-Methylation
Maintenance-Of-Location
Maturation-Of-SSU-RRNA
Maturation-Of-SSU-RRNA-From-Tricistronic-RRNA-Transciupt-SSU-RRNA-5-8S-RRNA-LSU-RRNA
Mitochondrial-Gene-Expression
Mitochondrial-Membrane-Organization
Mitochondrial-RNA-Metabolic-Process
Mitotic-DNA-Integrity-Checkpoint-Signaling
Mitotic-Sister-Chromatid-Segregation
Monoatomic-Cation-Transmembrane-Transport
Monoatomic-Cation-Tranport
Monoatomic-Ion-Transmembrane-Transport
Monoatomic-Ion-Transport
Monocarboxylic-Acid-Metabolic-Process
MRNA-Export-From-Nucleus
MRNA-Metabolic-Process
MRNA-Processing
MRNA-Transport
Negative-Regulation-Of-Apoptotic-Signaling-Pathway
Negative-Regulation-Of-Gene-Expression
Negative-Regulation-Of-Intrinsic-Apoptotic-Signaling-Pathway
Negative-Regulation-Of-Response-To-Endoplasmic-Reticulum-Stress
Neurogenesis
Neuron-Development
Non-Membrane-Bounded-Organelle-Assembly
Nuclear-Export
Nuclear-Transcribed-MRNA-Catabolic-Process
Nuclear-Transport
Nucleic-Acid-Catabolic-Process
Nucleobase-Containing-Compound-Transport
Nucleus-Organization
Organic-Acid-Metabolic-Process
Organic-Cyclic-Compound-Catabolic-Process
Organic-Hydroxy-Compound-Metabolic-Process
Organophosphate-Biosynthetic-Process
Organophosphate-Metabolic-Process
Phospholipid-Biosynthetic-Process
Phospholipid-Metabolic-Process
Positive-Regulation-of-DNA-Templated-Transcription-Initiation
Positive-Regulation-Of-Map-Kinase-Activity
Positive-Regulation-Of-MAPK-Cascade
Positive-Regulation-Of-MRNA-Catabolic-Process
Positive-Regulation-Of-MRNA-Metabolic-Process
Positive-Regulation-Of-Nervous-System-Development
Positive-Regulation-Of-Post-Translational-Protein-Modification
Positive-Regulation-Of-RNA-Metabolic-Process
Positive-Regulation-Of-Transcription-By-RNA-Polymerase-I
Positive-Regulation-Of-Translation
Post-Transcriptional-Regulation-of-Gene-Expression
Protein-Acetylation
Protein-Containing-Complex-Assembly
Protein-DNA-Complex-Assembly
Protein-DNA-Complex-Organization
Protein-Folding
Protein-Localization-To-Endoplasmic-Reticulum
Protein-Localization-To-Nucleus
Protein-RNA-complex-Organization
Regulation-Of-Alternative-MRNA-Splicing-Via-Spliceosome
Regulation-Of-Apoptotic-Signaling-Pathway
Regulation-Of-ATP-Dependent-Activity
Regulation-of-DNA-Replication
Regulation-Of-DNA-Templated-Transcription-Initiation
Regulation-Of-Hormone-Levels
Regulation-Of-Intrinsic-Apoptotic-Signaling-Pathway
Regulation-Of-Lymphocyte-Mediated-Immunity
Regulation-Of-MRNA-Catabolic-Process
Regulation-Of-MRNA-Metabolic-Process
Regulation-Of-MRNA-Processing
Regulation-Of-MRNA-Splicing-Via-Spliceosome
Regulation-Of-Nervous-System-Development
Regulation-Of-Neuron-Projection-Development
Regulation-Of-Response-To-Endoplasmic-Reticulum-Stress
Regulation-Of-RNA-Splicing
Regulation-Of-Transcription-By-RNA-Polymerase-I
Regulation-Of-Translation
Regulation-Of-Translation-Initiation
Regulatory-NCRNA-Mediated-Gene-Silencing
Response-To-Chemokine
Response-To-Endoplasmic-Reticulum-Stress
Response-To-Heat
Response-To-Topologically-Incorrect-Protein
Ribonucleoprotein-Complex-Biogenesis
Ribosomal-Large-Subunit-Biogenesis
Ribosomal-Small-Subunit-Biogenesis
Ribosome-Assembly
Ribosome-Biogenesis
RNA-Catabolic-Process
RNA-Export-From-Nucleus
RNA-Localization
RNA-Methylation
RNA-Modification
RNA-Polymerase-II-Preinitiation-Complex-Assembly
RNA-Processing
RNA-Splicing
RNA-Splicing-Via-Transesterification-Reactions
RRNA-Metabolic-Process
RRNA-Processing
Small-Molecule-Metabolic-Process
Spliceosomal-Complex-Assembly
Sterol-Metabolic-Process
Transcription-By-RNA-Polymerase-I
Transcription-Initiation-At-RNA-Polymerase-II-Promoter
Transcription-Preinitiation-Complex-Assembly
Translation
Translation-Initiation
Transmembrane-Transport
TRNA-Metabolic-process
TRNA-Processing
Vesicle-Mediated-Transport
p-val.
0.050
0.025
p 8
n 8
p 24
n 24
p 8
n 8
p 24
n 24
Classic Control
Pooled Control

Supplemental Figure S5: Time-dependent enrichment analysis performed with GSEA identifies up- or downregulated Gene Ontology biological processes terms with relevance for steatosis. On the left panel, identified pathways are shown based on the comparison of DEGs induced by steatotic compounds relative to untreated cells (classic control). On the right panel, identified pathways are shown based on the comparison of DEGs induced by steatotic compounds relative to untreated cells and cells treated with non-steatotic compounds (pooled control). Shown are heatmaps, colour coded according to p-value, with a selection of terms as defined by the adverse outcome pathway established for steatosis (coloured in red). p = positive enrichment score, n= negative enrichment score, time in hours.

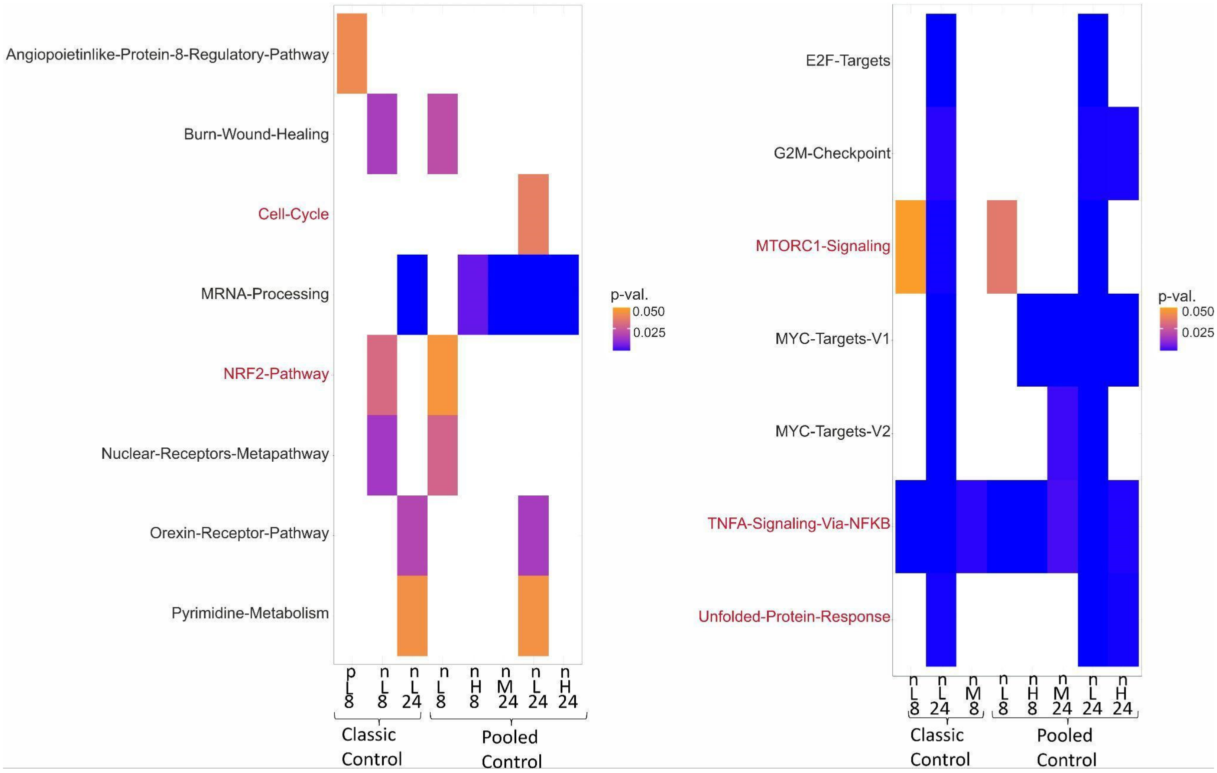

Supplemental Figure S6: Dose- and time-dependent enrichment analysis performed with GSEA identifies up- or downregulated terms with relevance for steatosis from the databases WikiPathways (left) and hallmark gene sets (right). For each, the left panel shows identified pathways based on the comparison of DEGs induced by steatotic compounds relative to untreated cells (classic control), while the right panel shows identified pathways based on the comparison of DEGs induced by steatotic compounds relative to untreated cells and cells treated with non-steatotic compounds (pooled control). Shown are heatmaps, colour coded according to p-value, with a selection of terms as defined by the adverse outcome pathway established for steatosis (coloured in red). p = positive enrichment score, n= negative enrichment score, L=low dose, M=middle dose, H=high dose, time in hours.

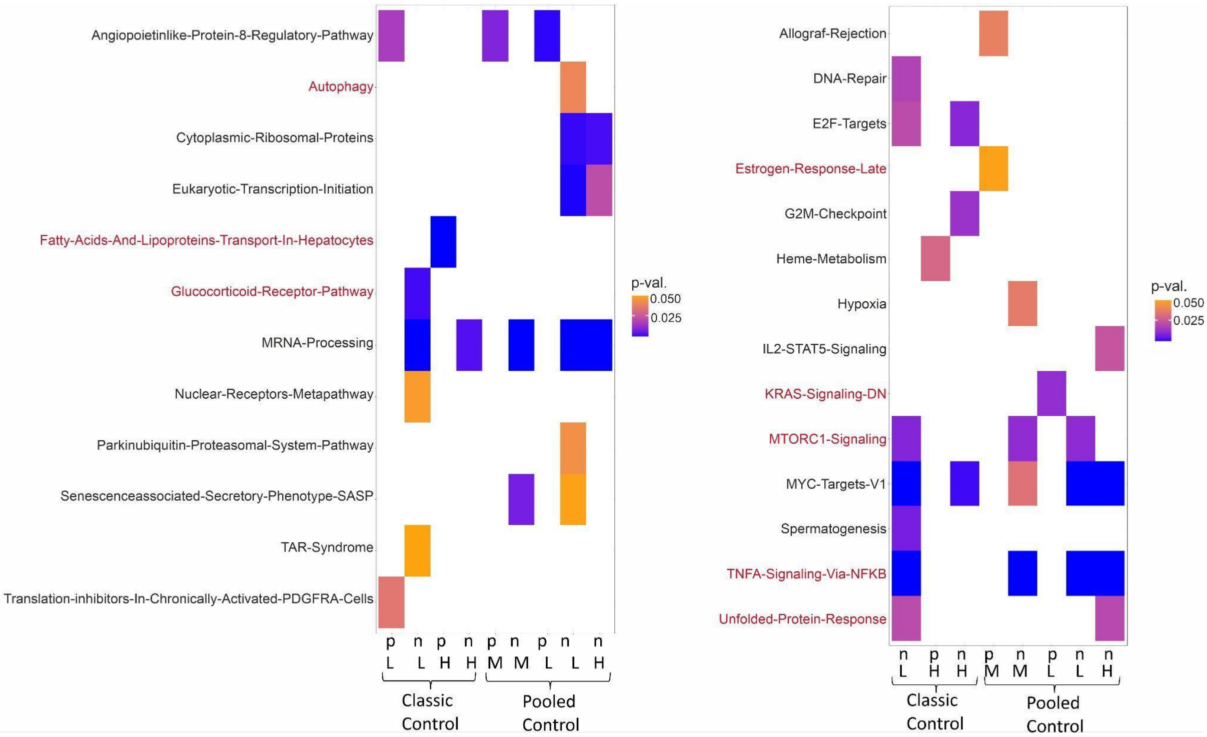

Supplemental Figure S7: Dose-dependent enrichment analysis performed with GSEA identifies up- or downregulated terms with relevance for steatosis from the databases WikiPathways (left) and hallmark gene sets (right). For each, the left panel shows identified pathways based on the comparison of DEGs induced by steatotic compounds relative to untreated cells (classic control), while the right panel shows identified pathways based on the comparison of DEGs induced by steatotic compounds relative to untreated cells and cells treated with non-steatotic compounds (pooled control). Shown are heatmaps, colour coded according to p-value, with a selection of terms as defined by the adverse outcome pathway established for steatosis (coloured in red). p = positive enrichment score, n= negative enrichment score, L=low dose, M=middle dose, H=high dose.

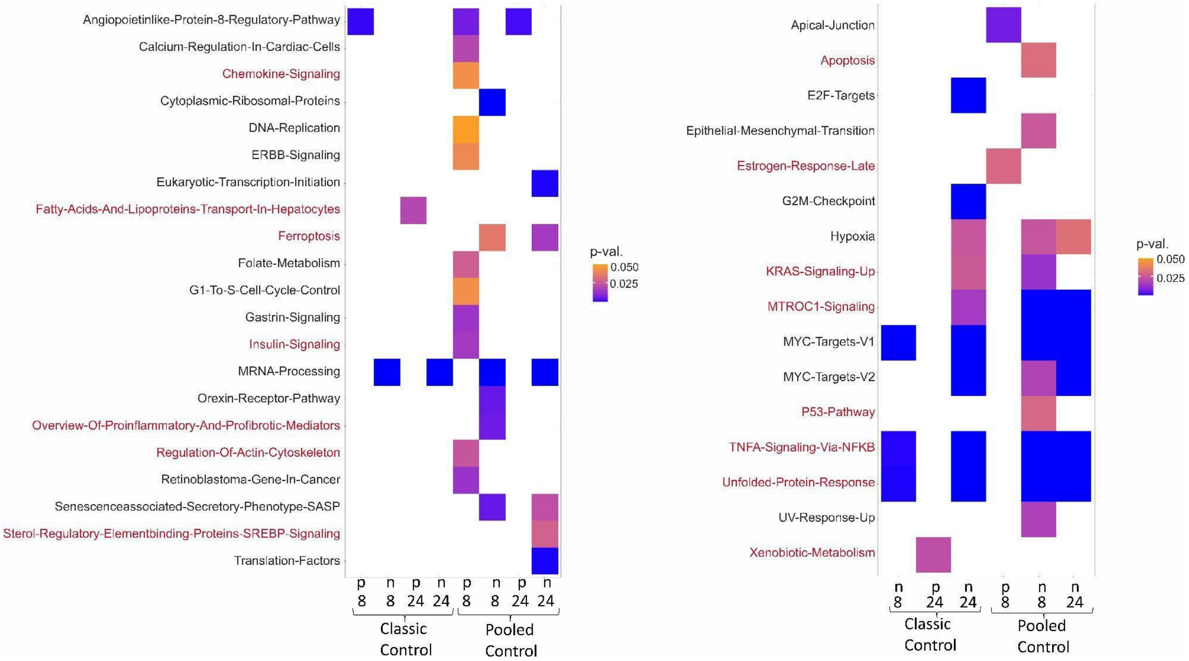

Supplemental Figure S8: Time-dependent enrichment analysis performed with GSEA identifies up- or downregulated terms with relevance for steatosis from the databases WikiPathways (left) and Hallmark gene sets (right). For each, the left panel shows identified pathways based on the comparison of DEGs induced by steatotic compounds relative to

untreated cells (classic control), while the right panel shows identified pathways based on the comparison of DEGs induced by steatotic compounds relative to untreated cells and cells treated with non-steatotic compounds (pooled control). Shown are heatmaps, colour coded according to p-value, with a selection of terms as defined by the adverse outcome pathway established for steatosis (coloured in red). p = positive enrichment score, n= negative enrichment score, time in hours.

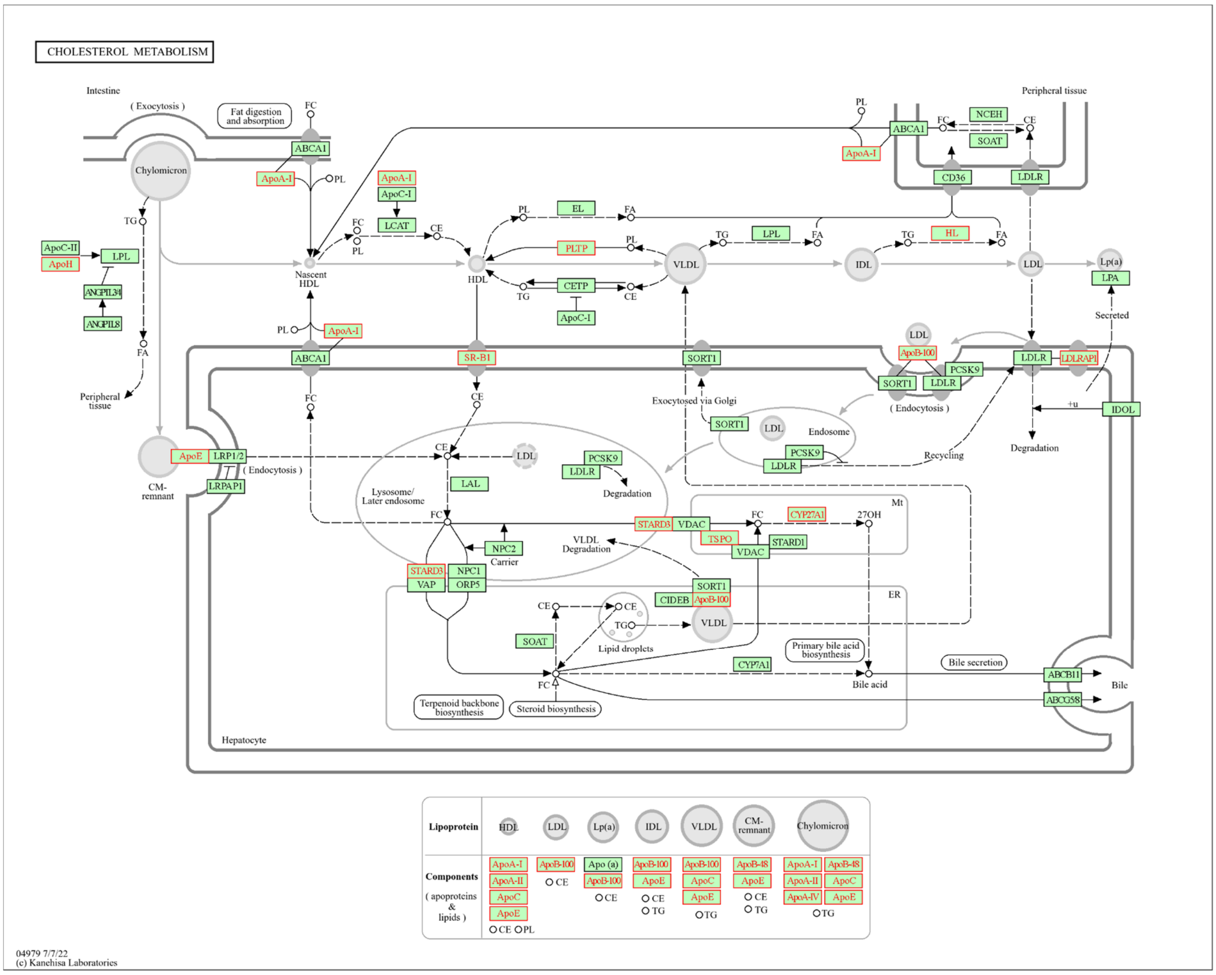

Supplemental Figure S9: Dose-dependent KEGG pathway analysis and DEVEA reveals cholesterol metabolism as a target of steatotic chemicals. In total, 3 data sets (doses: low, medium and high and all timepoints pooled) were used to identify pathways based on the comparison of DEGs induced by steatotic compounds relative to untreated cells and cells treated with non-steatotic compounds. Red boxes indicate up-regulated genes.

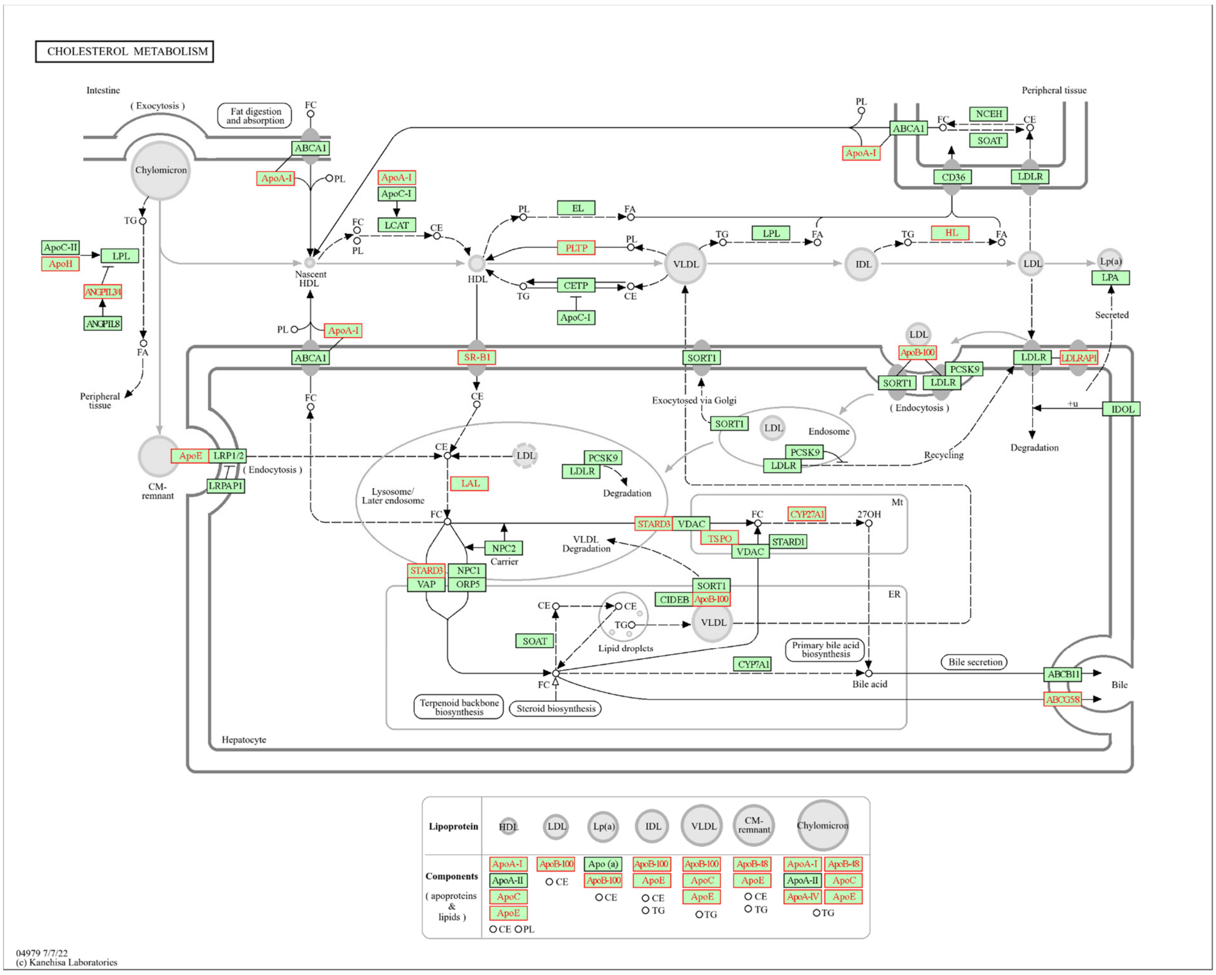

Supplemental Figure S10: Time-dependent KEGG pathway analysis and DEVEA reveals cholesterol metabolism as a target of steatotic chemicals. In total, 3 data sets (exposure time: 2, 8 and 24 hours and all doses pooled) were used to identify pathways based on the comparison of DEGs induced by steatotic compounds relative to untreated cells and cells treated with non-steatotic compounds. Red boxes indicate up-regulated genes.

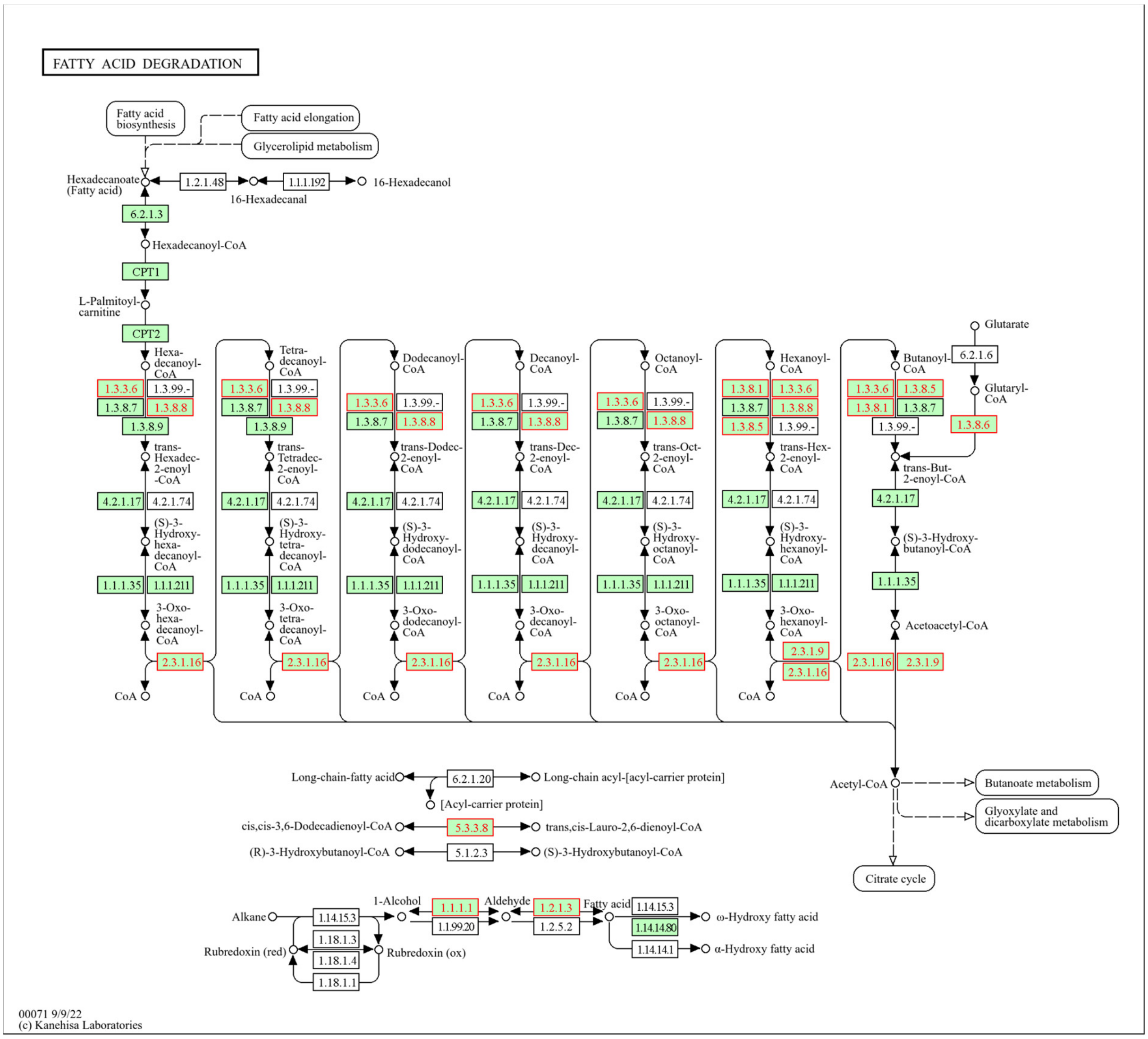

Supplemental Figure S11: Dose-dependent KEGG pathway analysis and DEVEA reveals glycerophospholipid metabolism as a target of steatotic chemicals. In total, 3 data sets (doses: low, medium and high and all timepoints pooled) were used to identify pathways based on the comparison of DEGs induced by steatotic compounds relative to untreated cells and cells treated with non-steatotic compounds. Red boxes indicate up-regulated genes.

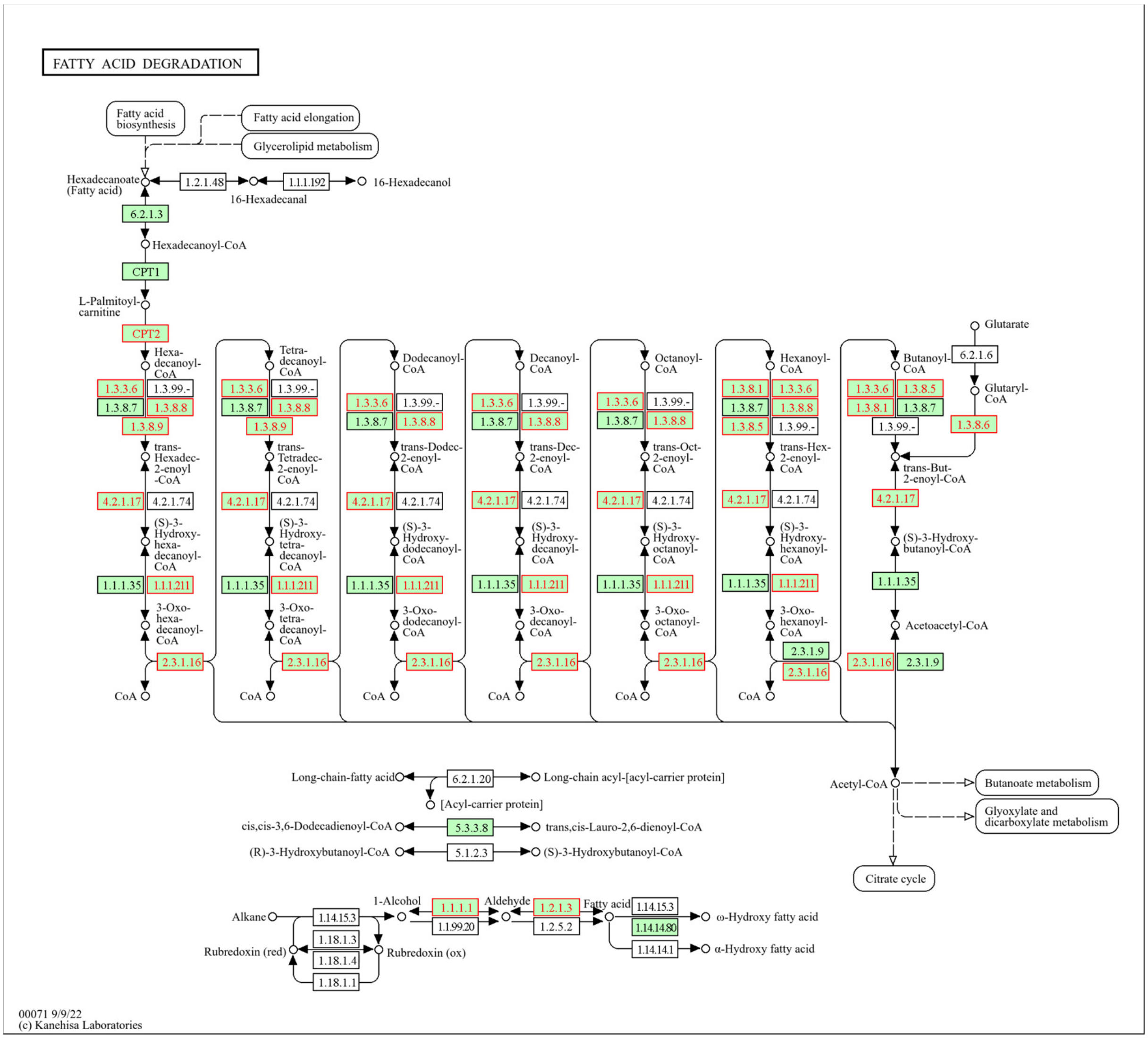

Supplemental Figure S12: Time-dependent KEGG pathway analysis and DEVEA reveals glycerophospholipid metabolism as a target of steatotic chemicals. In total, 3 data sets (exposure time: 2, 8 and 24 hours and all doses pooled) were used to identify pathways based on the comparison of DEGs induced by steatotic compounds relative to untreated cells and cells treated with non-steatotic compounds. Red boxes indicate up-regulated genes.

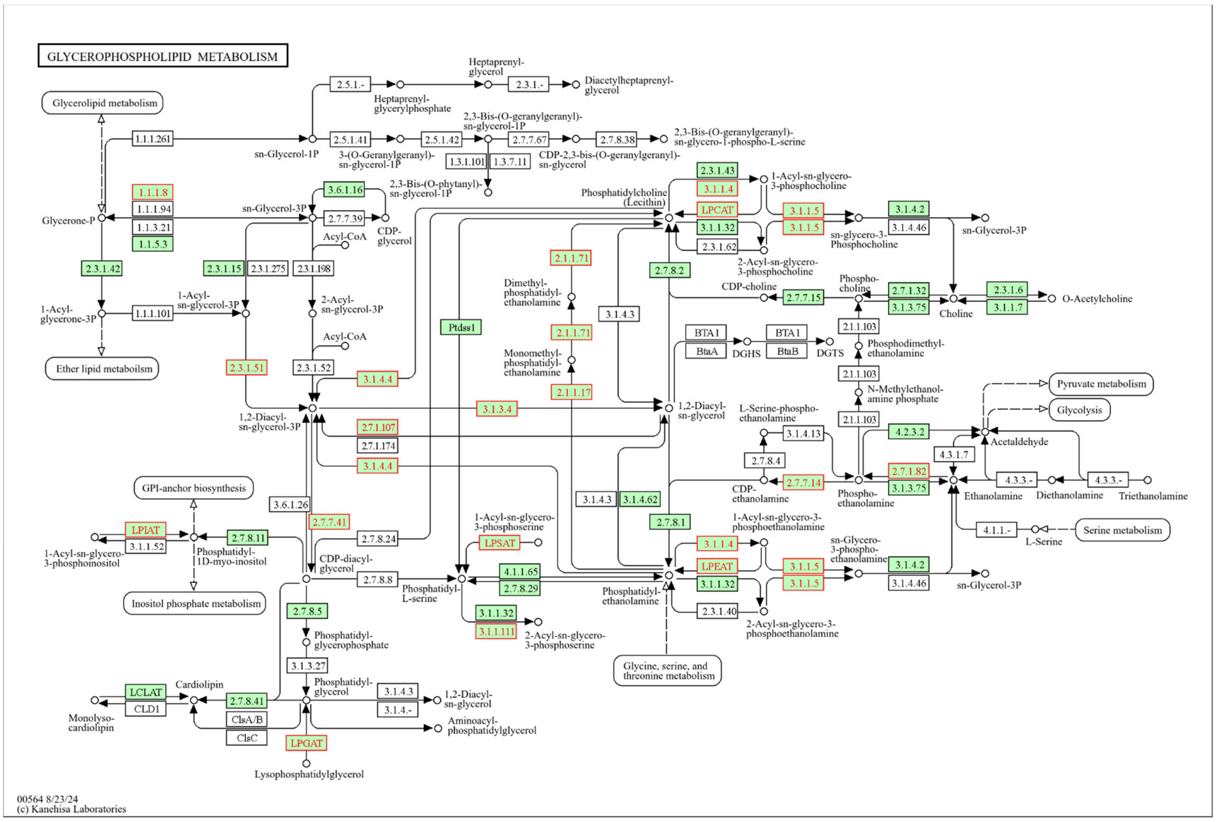

Supplemental Figure S13: Dose-dependent KEGG pathway analysis and DEVEA reveals fatty acid degradation as a target of steatotic chemicals. In total, 3 data sets (doses: low, medium and high and all timepoints pooled) were used to identify pathways based on the comparison of DEGs induced by steatotic compounds relative to untreated cells and cells treated with non-steatotic compounds. Red boxes indicate up-regulated genes.

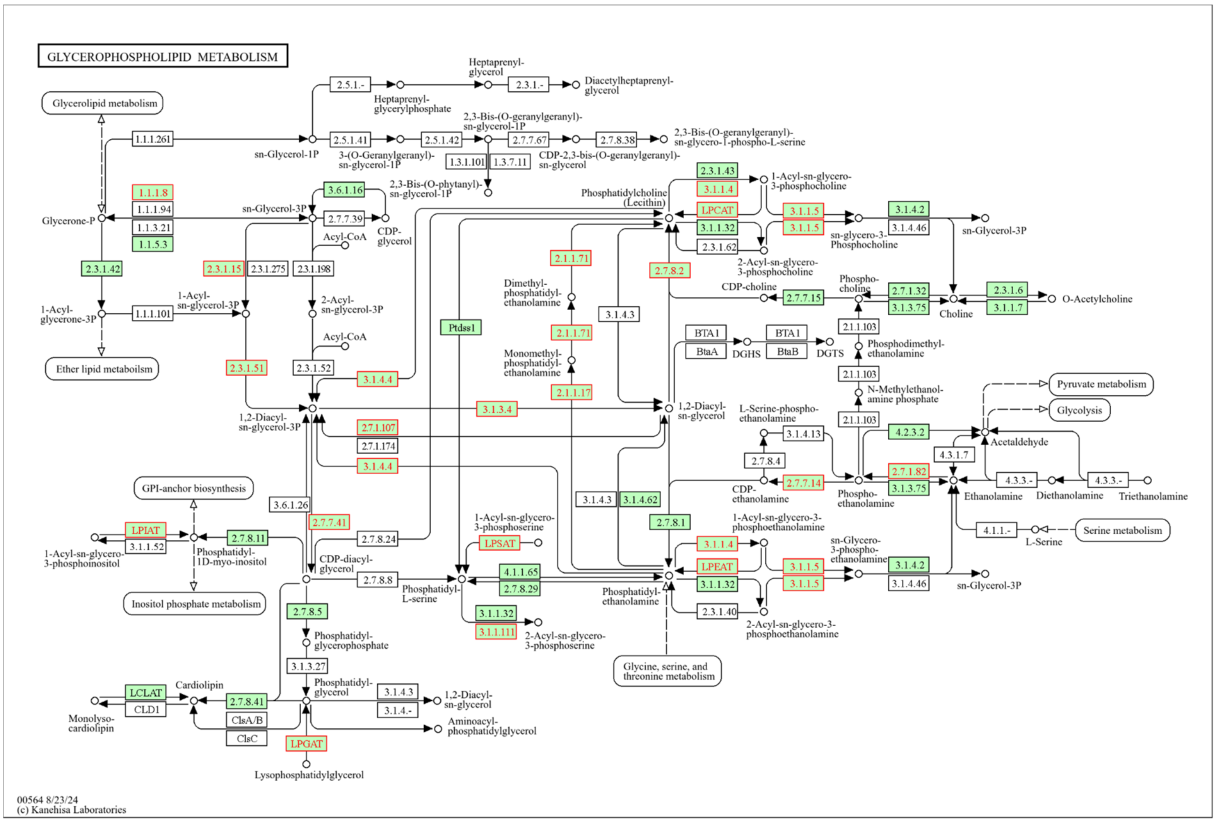

Supplemental Figure S14: Time-dependent KEGG pathway analysis and DEVEA reveals fatty acid degradation as a target of steatotic chemicals. In total, 3 data sets (exposure time: 2, 8 and 24 hours and all doses pooled) were used to identify pathways based on the comparison of DEGs induced by steatotic compounds relative to untreated cells and cells treated with non-steatotic compounds. Red boxes indicate up-regulated genes.

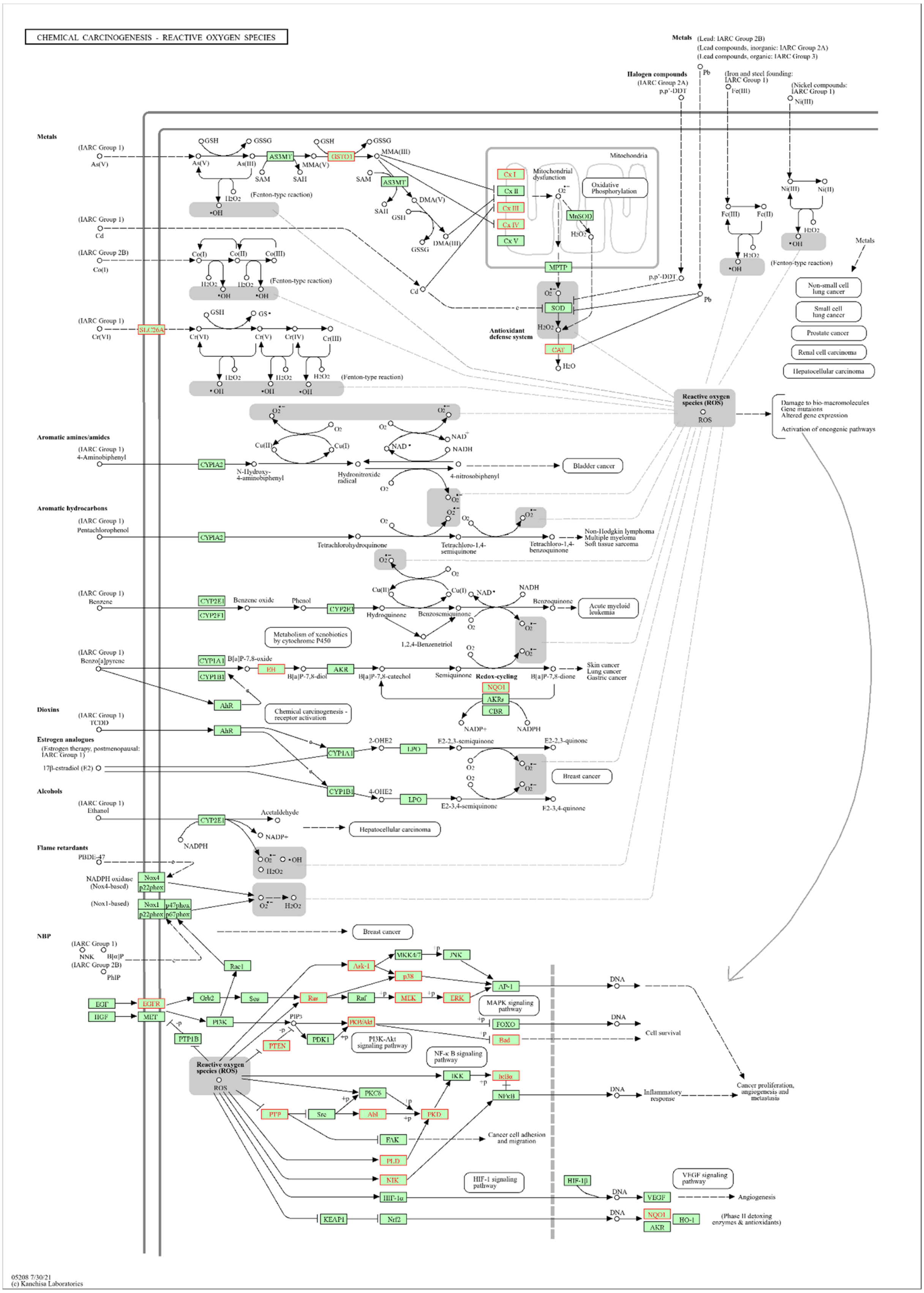

Supplemental Figure S15: Time-dependent KEGG pathway analysis and DEVEA reveals chemical carcinogenesis/ROS as a target of steatotic chemicals. In total, 3 data sets (exposure time: 2, 8 and 24 and all doses pooled) were used to identify pathways based on the comparison of DEGs induced by steatotic compounds relative to untreated cells and cells treated with non-steatotic compounds. Red boxes indicate up-regulated genes.

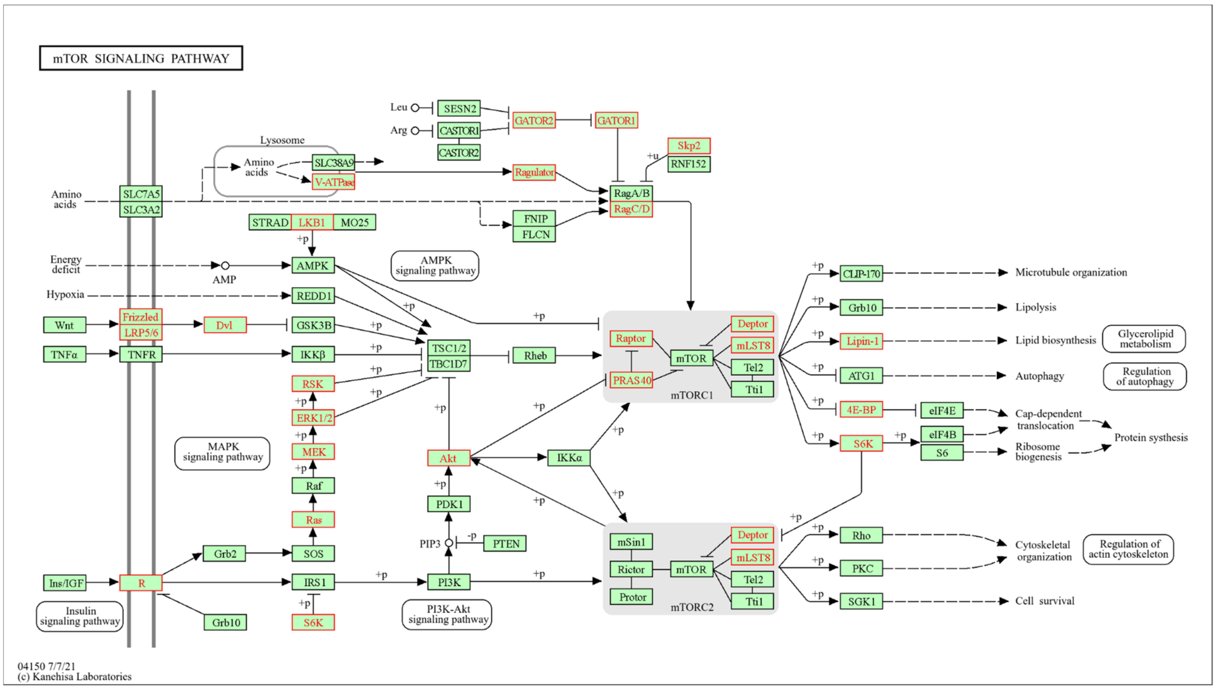

Supplemental Figure S16: Dose-dependent KEGG pathway analysis and DEVEA reveals the mTOR pathway as a target of steatotic chemicals. In total, 3 data sets (doses: low, medium and high and all timepoints pooled) were used to identify pathways based on the comparison of DEGs induced by steatotic compounds relative to untreated cells and cells treated with non-steatotic compounds. Red boxes indicate up-regulated genes.

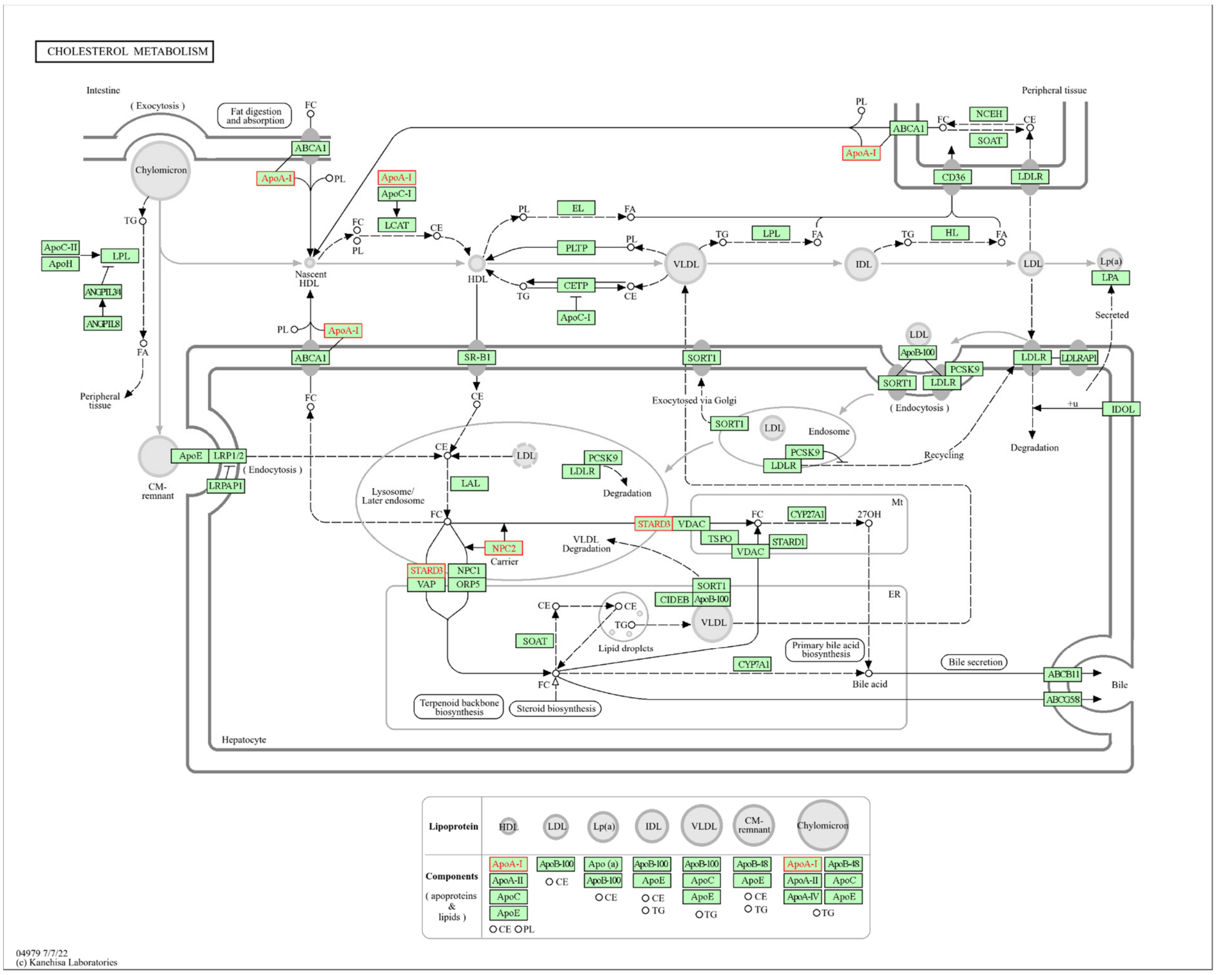

Supplemental Figure S17: Dose- and time-dependent KEGG pathway analysis of GSEA derived GO term Lipid Metabolic Processes reveals cholesterol metabolism as a target of steatotic chemicals. In total, 9 data sets (exposure time: 2, 8 and 24 hours; doses: low, medium and high) were used to identify pathways based on the comparison of DEGs induced by steatotic compounds relative to untreated cells. Red boxes indicate up-regulated genes.

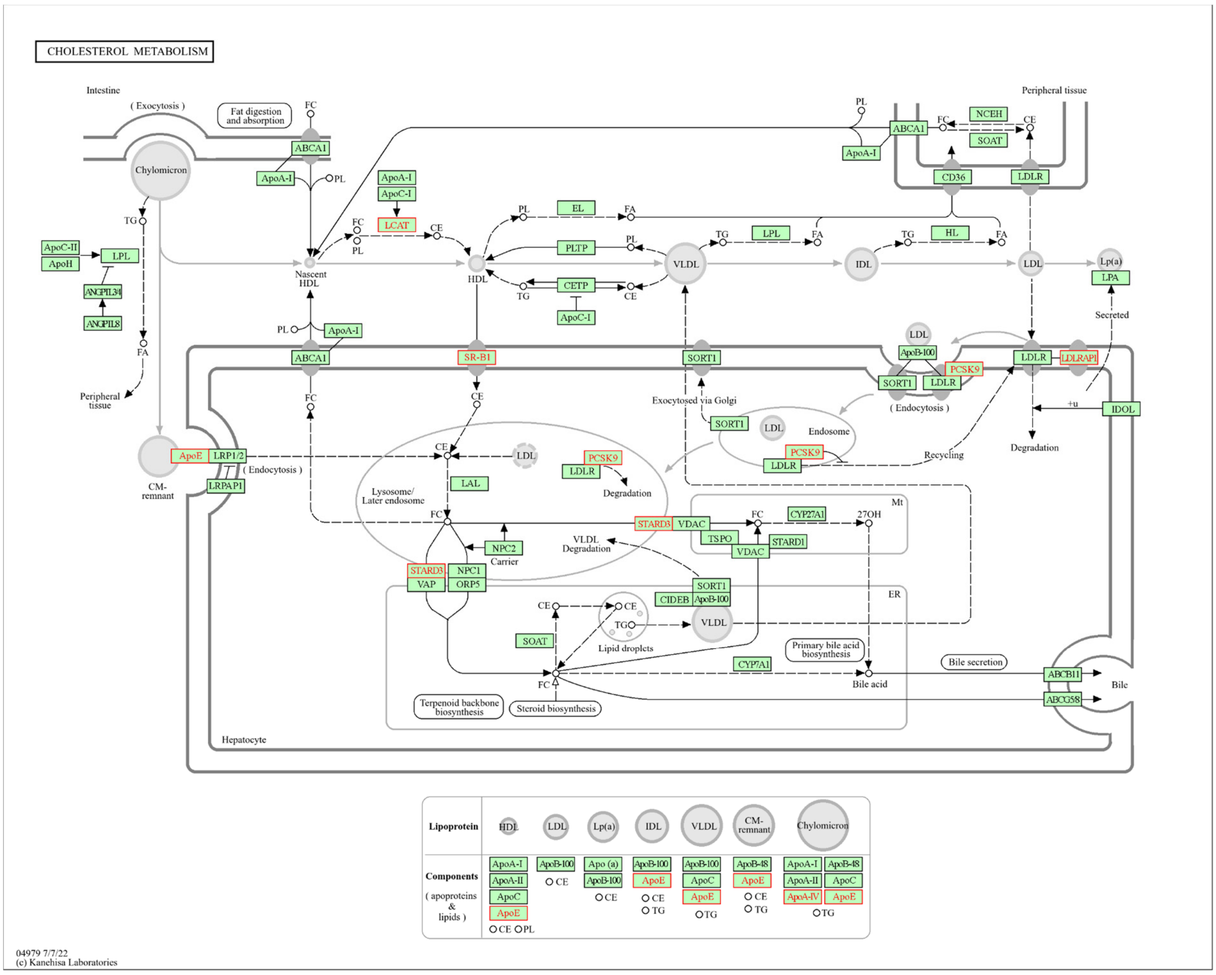

Supplemental Figure S18: Dose- and time-dependent KEGG pathway analysis of GSEA derived GO term Lipid Metabolic Processes reveals cholesterol metabolism as a target of steatotic chemicals. In total, 9 data sets (exposure time: 2, 8 and 24 hours; doses: low, medium and high) were used to identify pathways based on the comparison of DEGs induced by steatotic compounds relative to untreated cells and cells treated with non-steatotic compounds. Red boxes indicate up-regulated genes.

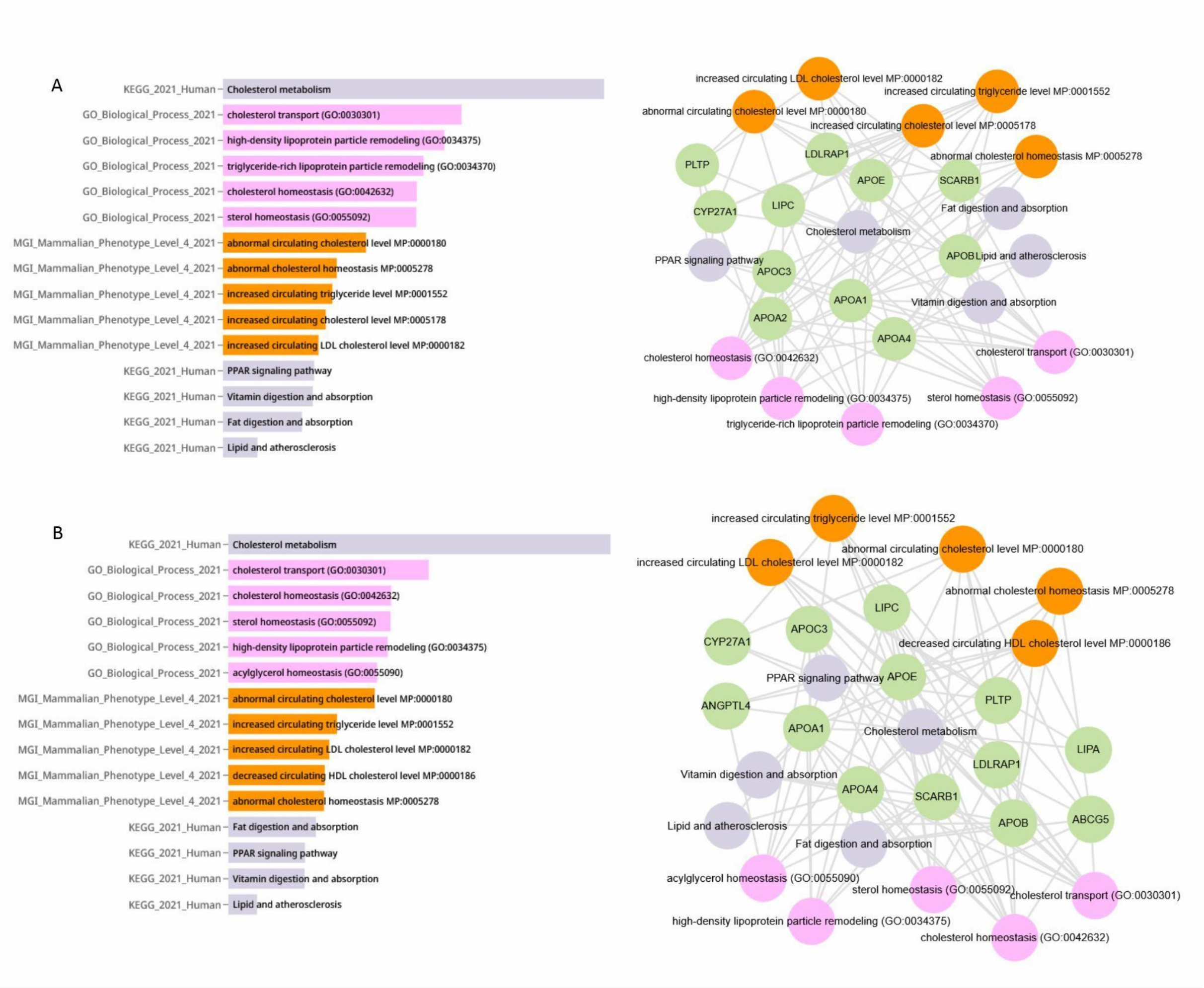

Supplemental Figure S19: Enrichr-KG analysis links gene sets deregulated by steatotic chemicals identified by DEVEA to cholesterol metabolism, associated biological processes and adverse mammalian phenotypes. A: Dose dependent analysis. In total, 3 analysis groups (doses: low, medium and high and all timepoints pooled) were used to identify DEGs induced by steatotic compounds relative to untreated cells and cells treated with non-steatotic compounds. Bar chart of enriched terms ordered by p-value and network view. Colour code: grey-KEGG terms, pink: GO-terms, orange: mammalian phenotypes, green: de-regulated genes. B: Analysis in dependence of time (exposure time: 2, 8 and 24 hours and all doses pooled).

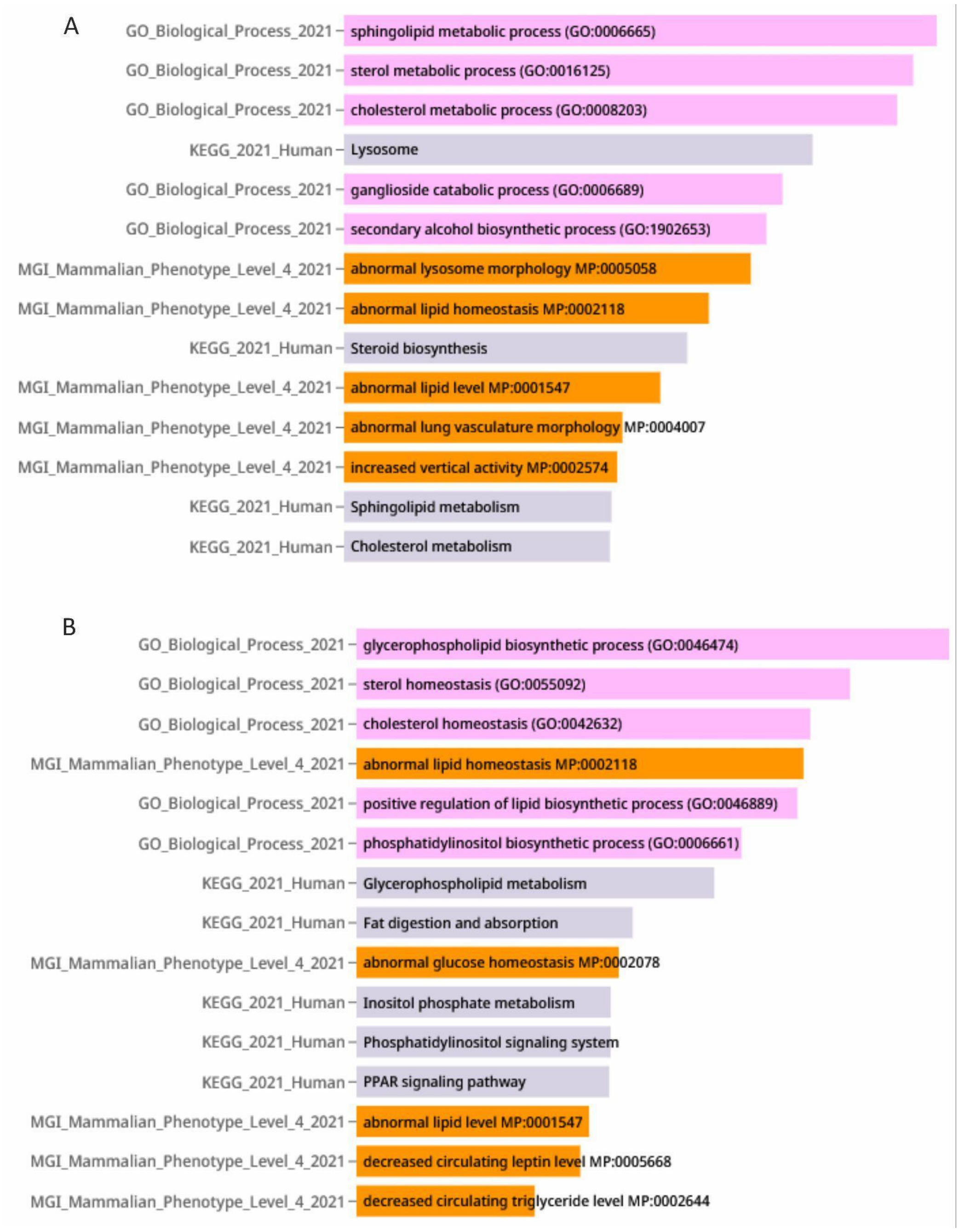

Supplemental Figure S20: Enrichr-KG analysis links genes indicated as deregulated by steatotic chemicals through GSEA analysis in GO term Lipid Metabolic Processes to associated biological processes and adverse mammalian phenotypes. In total, 9 analysis groups (exposure time: 2, 8 and 24 hours; doses: low, medium and high) were used to identify DEGs induced by steatotic compounds relative to untreated cells (classic control) (A) or relative to untreated cells and cells treated with non-steatotic compounds (pooled control) (B). A: Analysis relative to classic control. Bar chart of enriched terms ordered by p-value. Colour code: grey-KEGG terms, pink: GO-terms, orange: mammalian phenotypes, green: de-regulated genes. B: Analysis relative to pooled control. While both comparisons (A, B) show links to cholesterol and steroid metabolism, using the pooled control as a reference (B) further reveals positive regulation of lipid biosynthesis as well as PPAR signalling and fat digestion and absorption as targets.

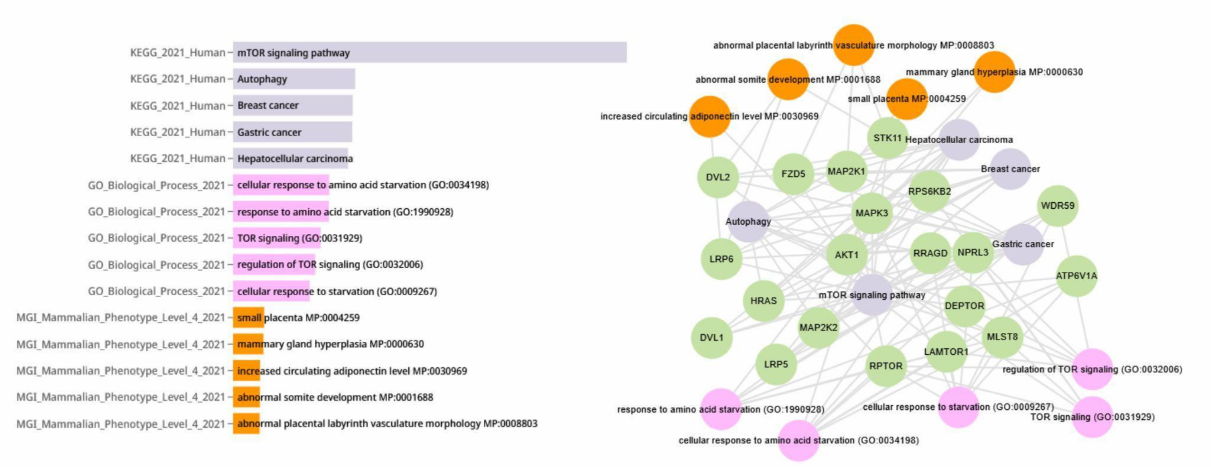

Supplemental Figure S21: Enrichr-KG analysis links dose-dependent gene sets deregulated by steatotic chemicals identified by DEVEA to mTOR signalling pathway, associated biological processes and adverse mammalian phenotypes. In total, 3 data sets (doses: low, medium and high and all timepoints pooled) were used to identify DEGs induced by steatotic compounds relative to untreated cells and cells treated with non-steatotic compounds. Bar chart of enriched terms ordered by p-value and network view. Colour code: grey-KEGG terms, pink: GO-terms, orange: mammalian phenotypes, green: de-regulated genes.

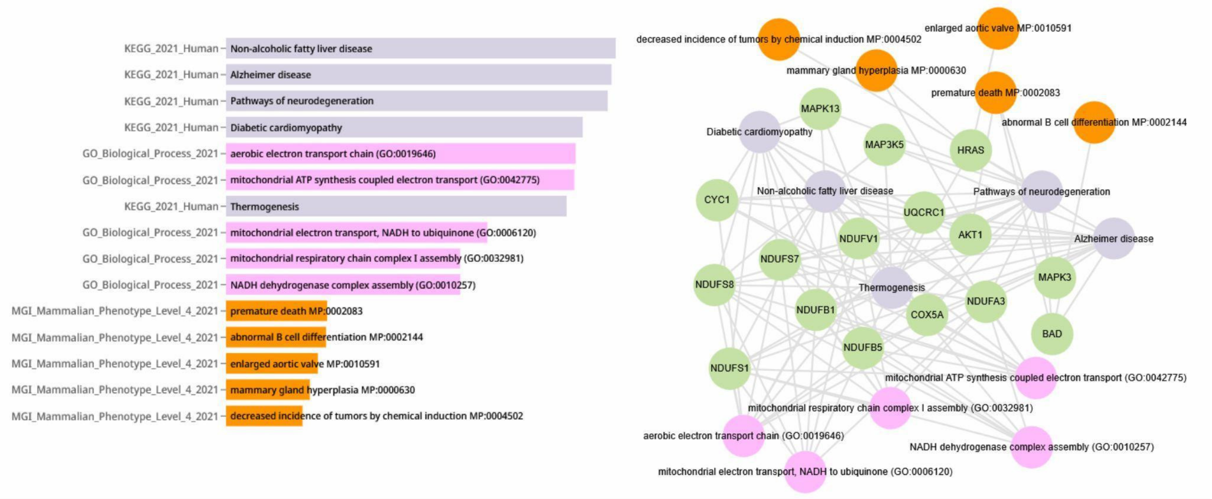

Supplemental Figure S22: Enrichr-KG analysis links time-dependent gene sets deregulated by steatotic chemicals identified by DEVEA to chemical carcinogenesis/ROS, associated biological processes and adverse mammalian phenotypes. In total, 3 data sets (exposure time: 2, 8 and 24 hours and all doses pooled) were used to identify DEGs induced by steatotic compounds relative to untreated cells and cells treated with non-steatotic compounds. Bar chart of enriched terms ordered by p-value and network view. Colour code: grey-KEGG terms, pink: GO-terms, orange: mammalian phenotypes, green: de-regulated genes.

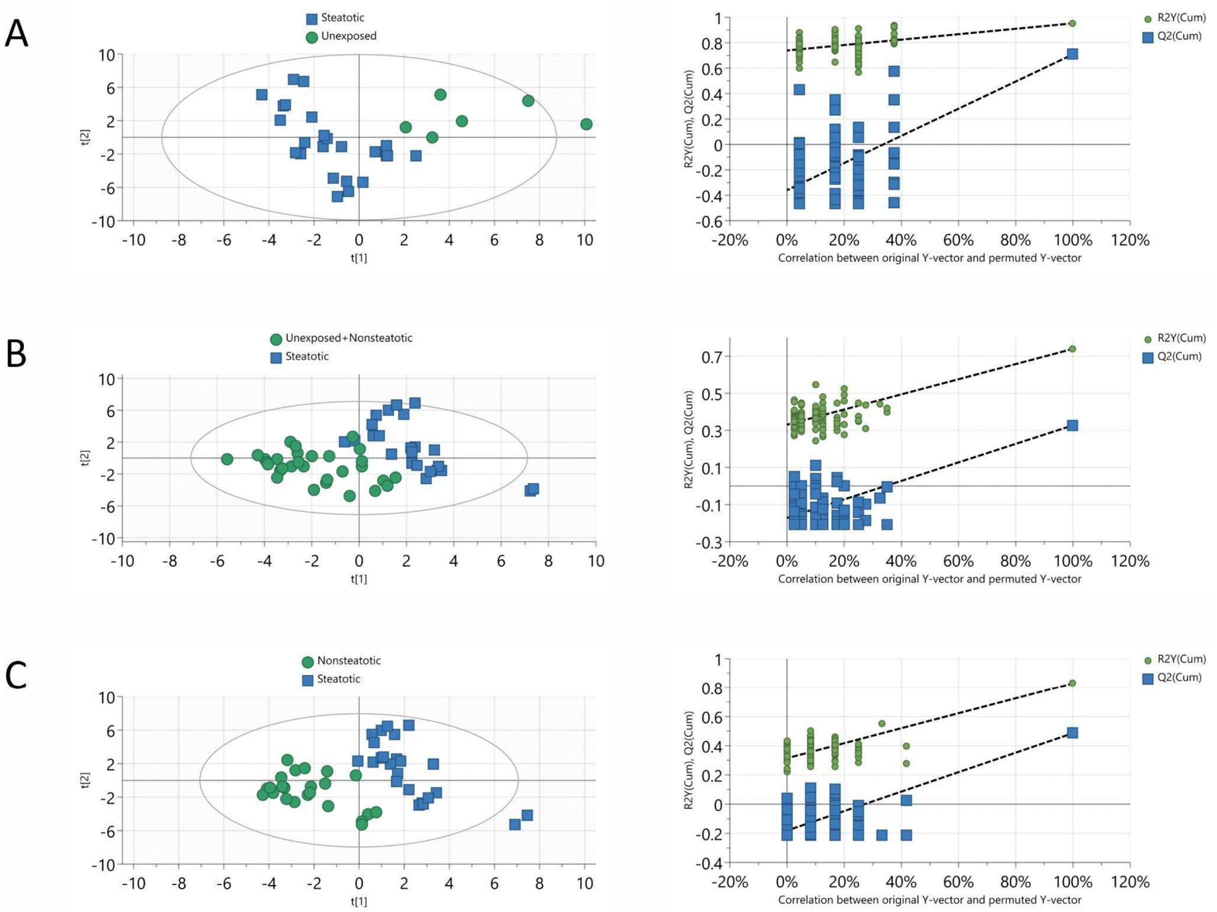

Supplemental Figure S23: Improving identification of metabolites associated with steatosis. A: PLS-DA model comparing cells treated with steatotic chemicals versus unexposed cells, score plot and permutation plot. Model metrics: RY(cum) = 0.95, Q(cum) = 0.71. P-value of cross validation = 0.006. Permutation metrics: R2=(0.0, 0.739), Q2=(0.0, -0.36). B: PLS-DA model comparing cells treated with steatotic chemicals versus unexposed cells and cells treated with non-steatotic chemicals, score plot and permutation plot. Model metrics: RY(cum) = 0.74, Q(cum) = 0.33. P-value of cross validation < 0.001. Permutation metrics: R2=(0.0, 0.739), Q2=(0.0, -0.36). C: PLS-DA model comparing cells treated with steatotic chemicals versus cells treated with non-steatotic chemicals, score plot and permutation plot. Model metrics: RY(cum) = 0.83, Q(cum) = 0.49. P-value of cross validation < 0.001. Permutation metrics: R2=(0.0, 0.315), Q2=(0.0, -0.181).
